# Efficacy of Music Intervention for Dental Anxiety Disorders: A Systematic Review and Meta-Analysis

**DOI:** 10.3390/medicina59020209

**Published:** 2023-01-20

**Authors:** Kui Tan, Hao Liu, Shuang Huang, Conghua Li

**Affiliations:** 1 Stomatological Hospital of Chongqing Medical University, Chongqing Medical University, Chongqing 401147, China; 2 Chongqing Key Laboratory of Oral Diseases and Biomedical Sciences, Chongqing 401147, China; 3 Chongqing Municipal Key Laboratory of Oral Biomedical Engineering of Higher Education, Chongqing 401147, China; 4 Department of Oral and Maxillofacial Surgery, The First Affiliated Hospital of Chongqing Medical University, Chongqing 400010, China

**Keywords:** dental anxiety disorder, music, meta-analysis

## Abstract

*Objective* To evaluate the effectiveness of music therapy for dental anxiety disorders. *Methods* In order to gather clinical randomized controlled trials comparing the effectiveness of music interventions to traditional oral manipulation in patients with dental anxiety disorders, computer searches of the electronic databases of Wanfang, CNKI, VIP, PubMed, Web of Science, ScienceDirect, Cochrane library, Scopus, and CINAHL were conducted. The search period covered from 23 December 2022, through to the development of the database. The Cochrane Handbook was used to assess the quality of the included literature, and two researchers independently conducted the literature screening and data extraction. Stata 17.0 and RevMan 5.3 were used to conduct the meta-analysis. *Results* The preoperative baseline levels of the music intervention group were similar to those of the control group (*p* > 0.05), according to the meta-analysis, and music intervention significantly decreased heart rate (I^2^ = 81.2%, WMD (95% CI): −7.33 (−10.07, −4.58), *p* < 0.0001), systolic blood pressure fluctuations (I^2^ = 85.6%, WMD (95% CI): −6.10(−9.25, 2.95), *p* < 0.0001), diastolic blood pressure (I^2^ = 79.7%, WMD (95% CI): −4.29(−6.57, −2.02), *p* < 0.0001) fluctuations, anxiety scores (I^2^ = 19.6%, WMD (95% CI): −9.04(−11.45, 6.63), *p* < 0.0001), and pain scores (I^2^ = 32.7%, WMD (95% CI): −7.64(−9.43, −5.85), *p* < 0.0001), as well as significantly lowered anxiety and pain levels and raised patients’ cooperation rates (I^2^ = 0%, OR (95% CI): 3.03(1.24, 7.40), *p* = 0.02). *Conclusions* Music interventions are effective for dental anxiety disorders, but given the limitations of the study, more multicenter, large-sample, high-quality randomized controlled trials are needed to further validate the findings and obtain more objective and reliable clinical evidence.

## 1. Introduction

Dental anxiety disorder is a collection of fears related to therapeutic or preventative dental procedures, which are frequently exhibited by extreme tension, lack of cooperation, and even trembling, nausea, and resistance during the patient’s visit [1,2]. Dental anxiety affects 15.3% of adults on average [3], and can affect anywhere between 6% and 75% of children or adolescents [4]. Patients with dental anxiety disorder frequently avoid going to the dentist due to anxiety or dread, or they are excessively scared while there, which results in a bad visit experience, which in turn increases the fear and results in refusal to go to the dentist in a vicious cycle [5,6,7]. As a result, dental anxiety negatively impacts patients’ physical and mental health. However, there are currently no well-defined treatments or controls for dental anxiety disorder, and additional research is urgently required to make treatment more accessible.

The major ways that musical interventions reduce stress, discomfort, anxiety, and loneliness in patients are to improve their mood, behavior, and quality of life [8]. Numerous studies conducted in recent years [9,10,11,12,13,14,15] have demonstrated the value of music interventions in the treatment of serious illnesses, depression, schizophrenia, anxiety disorders, and childhood autism. With the gradual growth of music therapy, its range of applications has expanded, and in recent years, some studies have suggested that music treatments used in conjunction with traditional oral care can significantly relieve the symptoms of patients with dental anxiety disorder. The effectiveness of music intervention in the treatment of dental anxiety disorders has not been fully elucidated, however, and the current study has limitations including a small sample size, dispersed study locations, and inconsistent outcomes. By using meta-analysis, this study aims to assess the clinical efficacy of music intervention therapy in supporting dental anxiety patients during normal treatment procedures and to serve as a guide for clinical practice.

## 2. Materials and Methods

### 2.1. Search Strategy

We performed a computer search of electronic databases, including Wanfang, China National Knowledge Infrastructure (CNKI), China Science and Technology Journal Database (VIP), Pubmed, Web of Science (WOS), ScienceDirect, Cochrane Library, Scopus and CINAHL. The search terms were “oral”, “dental”, “music”, etc. Additionally, manual searches were conducted on ClinicalTrials.gov, the Chinese Clinical Trial Registry (ChiCTR), and all references that were to be included in the literature. The detailed search strategy is shown in Supplementary File S1 (Keywords and search result).

### 2.2. Inclusion Criteria

Table 1 displays the inclusion and exclusion standards. We looked for any studies that included music interventions with traditional oral care rather than just traditional oral care. The investigations all fell under the category of clinical randomized controlled trials (RCTs). Studies without full text access, retrospective studies, case reports, animal studies, and reviews were not included. Additionally, any patient data that overlapped were eliminated when data from two or more studies or samples were published repeatedly. This method criteria and operations were carried out separately by various people.

### 2.3. Data Extraction and Quality Assessment

Data were independently extracted by two authors (K.T. and H.L.), and discrepancies were settled through discussion. The consistency and validity of the literature researchers were pre-tested as good and reliable with no researcher bias (Kappa = 0.68, Supplementary File S5). The authors of the study, the year that the study was published, the nation, the population, the disease of the subjects in the study, the sample size of the experimental and control groups, the gender distribution of the sample, the mean age, the mean and standard deviation of the preoperative and postoperative heart rates, the pain scores, the MDAS, CFSS-DS, and S-AI, and the cooperation rates of the experimental and control groups were all extracted.

Two separate reviewers independently evaluated the included studies’ quality using the Cochrane handbook. Each RCT was assessed for its randomization, allocation concealment, blinding administration, outcome data, report selection, and other biases. Low risk, uncertain risk, and high risk were the three levels of examination. When reviewers could not agree, a conversation was used to decide the rating. High-quality studies were given more of a low-risk label, but studies were also adopted in a controlled manner when the risk was manageable.

### 2.4. Statistical Analyses

For continuous variables, the weighted mean difference (WMD) was used as the effect indicator, while the ratio of ratios (OR) was used for dichotomous variables. For each effect size calculation, 95% confidence intervals (CI) were employed. When *p* ≥ 0.1 and I^2^ ≤ 50%, the heterogeneity of the test results was considered to be small, and a fixed-effect model was used for merging; when *p* < 0.1 and I^2^ > 50%, the heterogeneity of the test results was considered to be large, and a random-effect model was used for merging. Additionally, a sub-group analysis or sensitivity analysis was carried out by excluding the literature on an individual basis. Descriptive evaluation was used if meta-analysis of the data was not possible. In order to examine the potential publication bias of included research, the Egger and Begger tests were performed. Statistics were judged significant when the difference was *p* < 0.05.

### 2.5. Statement

The Preferred Reporting Items for Systematic Reviews and Meta-Analyses (PRISMA) were followed when conducting this study [16]. The study’s software, which included Stata, RevMan, and Endnote, conformed with all applicable operational criteria. The independence and impartiality guiding principles were upheld throughout the execution of the study. Unbiased by the researcher, an impartial ethics and equity watchdog oversaw the entire study.

## 3. Results

### 3.1. Identification of Studies

**Figure 1:**
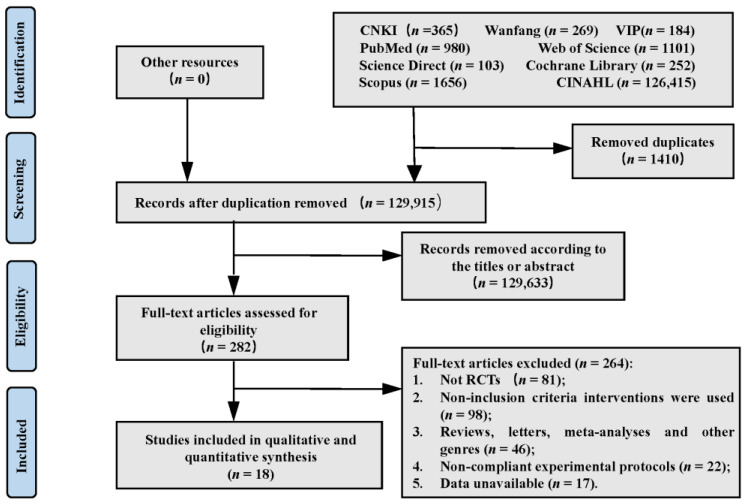
Characteristics of Included Studies.

Figure 1 displays our study’s selection procedure and results. The electronic literature search identified 131,325 articles and the manual search identified 0 articles. 1410 duplicate articles were removed. After checking the titles and abstracts of the remaining 129,633 articles, 282 articles were moved to the next selection stage. On the basis of the inclusion and exclusion criteria, 264 further items from this group were eliminated. The final 18 articles were included and subjected to meta-analysis.

**Figure 2:**
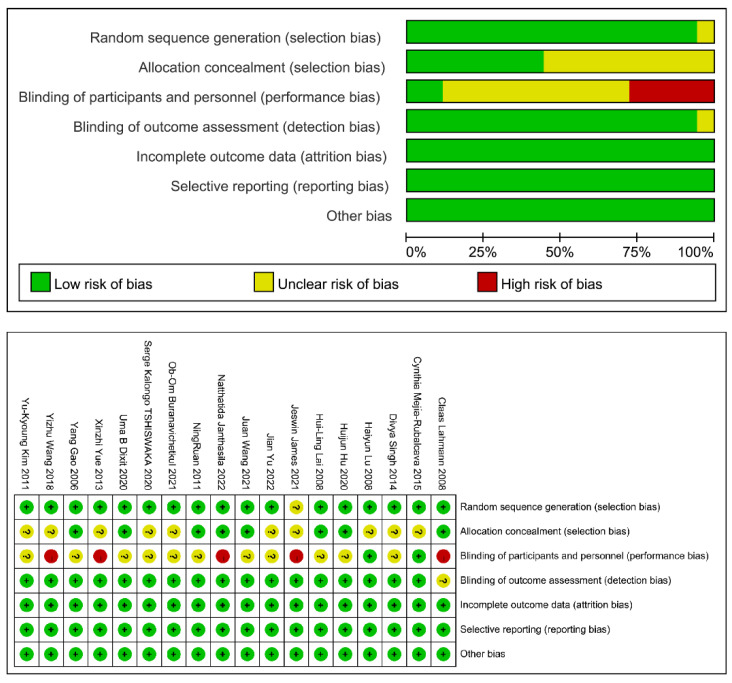
Risk bias assessment of the included studies [17,18,19,20,21,22,23,24,25,26,27,28,29,30,31,32,33,34].

Table 2 summarizes the characteristics of the six studies included in this review, including country, population, oral manipulation, sample size, age, and outcome. The studies were reported from May 2006 to December 2022 and were all single-center studies. A total of 1386 patients were included in the included literature, of which 690 received music therapy and conventional oral manipulation (experimental group) and 696 received conventional oral manipulation only (control group). Of these, seven studies [1,4,10,14,15,16,17] were conducted in children, 10 studies [2,3,5,6,8,9,11,12,13,18] were conducted in adults, and one study [7] did not specify the study population. The bias assessment based on the Cochrane handbook [16] considered the allocation blinding of 5 studies to be questionable and deemed “high risk”. No other significant bias was found, and the overall quality of the article was reliable (Figure 2).

### 3.2. Study Outcome

Supplementary File S2 summarizes the results of each of the included studies.

#### 3.2.1. Heart Rate

**Figure 3:**
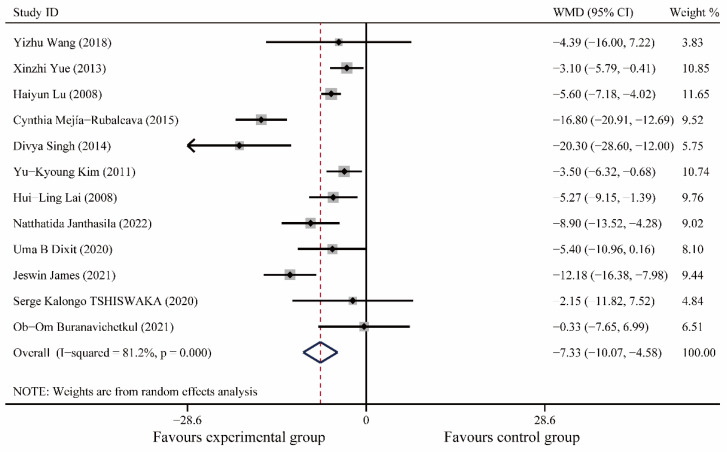
Forest plot of postoperative heart rate [20,21,24,25,26,27,29,30,31,32,33,34].

A total of 12 articles [20,21,24,25,26,27,29,30,31,32,33,34], including 1028 patients, were included. Meta-analysis showed no significant difference in heart rate between the experimental and control groups preoperatively (I^2^ = 39.5%, WMD (95% CI): −0.98(−2.07, 0.12), *p* = 0.08); postoperatively, music therapy significantly reduced heart rate in patients with dental anxiety (I^2^ = 81.2%, WMD (95% CI): −7.33(−10.07, 4.58), *p* < 0.0001), and further meta-regression showed that different study populations (children or adults, *p* = 0.32), different oral operations (*p* = 0.32), different countries (*p* = 0.13), and different years (*p* = 0.32) did not significantly affect the results of this study (Figure 3, Supplementary Files S2–S4).

#### 3.2.2. Systolic Blood Pressure

**Figure 4:**
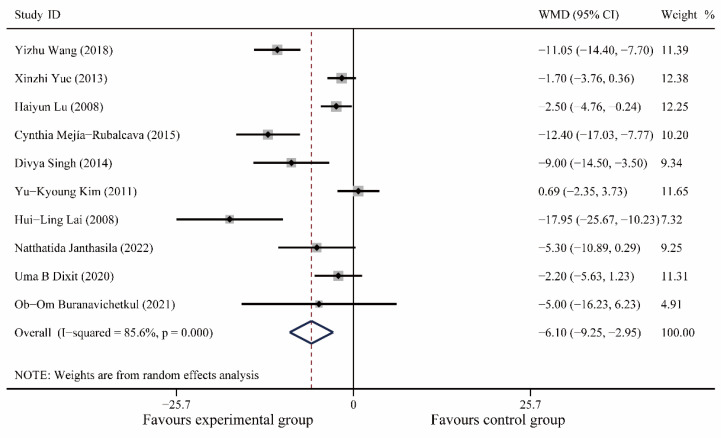
Forest plot of postoperative systolic blood pressure [20,21,24,25,26,27,29,30,31,34].

A total of 10 articles [20,21,24,25,26,27,29,30,31,34], including 888 patients, were included. Meta-analysis showed no significant difference in heart rate between the experimental and control groups preoperatively (I^2^ = 14.9%, WMD (95% CI): −0.23(−1.36, 0.89), *p* = 0.69); postoperatively, music therapy significantly reduced heart rate in patients with dental anxiety (I^2^ = 85.6%, WMD (95% CI): −6.10(−9.25, 2.95), *p* < 0.0001) and further meta-regression showed that different study populations (children or adults, *p* = 0.10), different oral operations (*p* = 0.32), different countries (*p* = 0.32), and different years (*p* = 0.32) did not significantly affect the results of this study (Figure 4, Supplementary Files S3 and S4).

#### 3.2.3. Diastolic Blood Pressure

**Figure 5:**
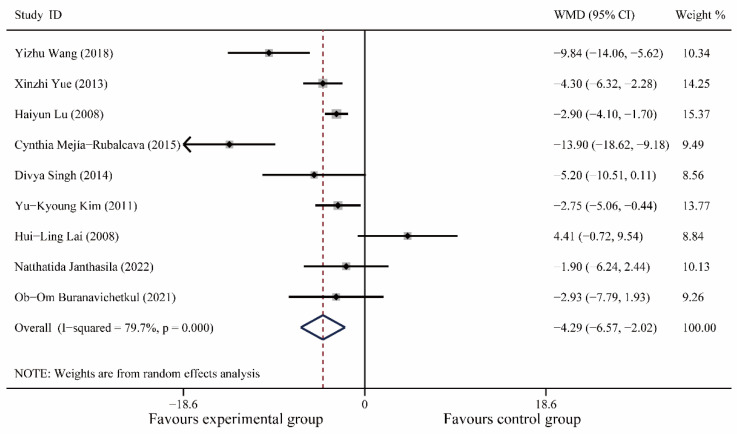
Forest plot of postoperative diastolic blood pressure [20,21,24,25,26,27,29,30,34].

A total of 9 articles [20,21,24,25,26,27,29,30,34], including 808 patients, were included. Meta-analysis showed no significant difference in heart rate between the experimental and control groups preoperatively (I^2^ = 66.7%, WMD (95% CI): −0.67(−2.66, 1.32), *p* = 0.51); postoperatively, music therapy significantly reduced heart rate in patients with dental anxiety (I^2^ = 79.7%, WMD (95% CI): −4.29(−6.57, −2.02), *p* < 0.0001) and further meta-regression showed that different study populations (children or adults, *p* = 0.15), different oral operations (*p* = 0.32), different countries (*p* = 0.32), and different years (*p* = 0. 35) did not significantly affect the results of this study (Figure 5, Supplementary Files S3 and S4).

#### 3.2.4. Pain Scores

**Figure 6:**
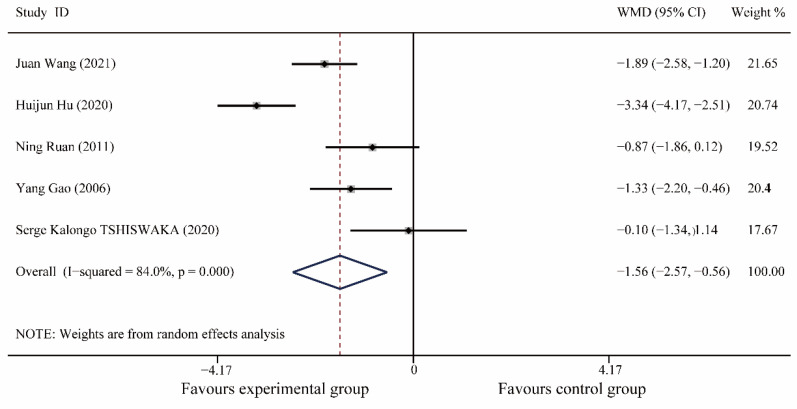
Forest plot of postoperative pain scores [18,19,22,23,33].

A total of 5 articles [18,19,22,23,33], including 280 patients, were included. Meta-analysis results showed no significant difference in pain scores between the experimental and control groups preoperatively (I^2^ = 0%, WMD (95% CI): 0.19(−0.27, 0.66), *p* = 0.41); postoperatively, music therapy significantly reduced pain scores in patients with dental anxiety (I^2^ = 84.0%, WMD (95% CI): −1.56(−2.57, −0.56), *p* < 0.0001) and further meta-regression showed that different study populations (children or adults, *p* = 0.32), different oral operations (*p* = 0.13), different countries (*p* = 0.07), and different years (*p* = 0. 32) did not significantly affect the results of this study (Figure 6, Supplementary Files S3 and S4).

#### 3.2.5. MDAS

A total of 3 articles [18,19,22], including 198 patients, were included. The results showed no significant difference in MDAS between patients in the experimental and control groups before surgery (I^2^ = 0%, WMD (95% CI): 0.14(−0.43, −0.71), *p* = 0.63); postoperatively, music therapy significantly reduced MDAS in patients with dental anxiety (I^2^ = 0%, WMD (95% CI): −2.96(−3.65, −2.27), *p* < 0.0001, Supplementary File S3).

#### 3.2.6. CFSS-DS

A total of 2 articles were included [17,30], including 125 patients. Meta-analysis showed no significant difference in CFSS-DS between patients in the preoperative experimental and control groups (I^2^ = 0%, WMD (95% CI): 0.49(−1.67, 2.65), *p* = 0.66); postoperatively, music therapy significantly reduced CFSS-DS in patients with dental anxiety (I^2^ = 19.6%, WMD (95% CI): −9.04(−11.45, 6.63), *p* < 0.0001, Supplementary File S3).

#### 3.2.7. S-AI

**Figure 7:**
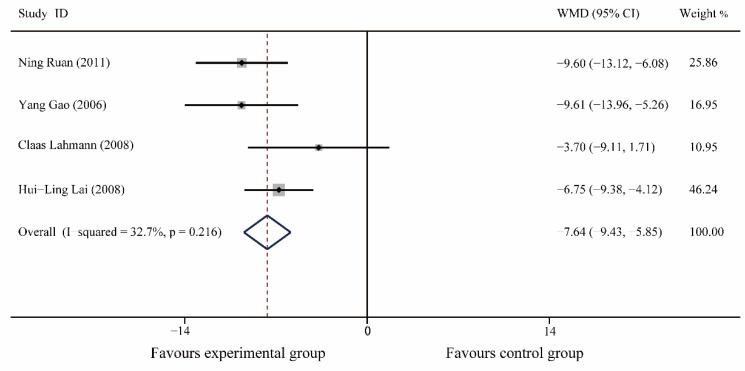
Forest plot of S-AI.

A total of 4 articles were included [22,23,28,29], including 224 patients. The results of the meta-analysis showed no significant difference in S-AI between the preoperative experimental and control patients (I^2^ = 0%, WMD (95% CI): 0.82(−0.73, 2.37), *p* = 0.30); postoperatively, music therapy significantly reduced S-AI in patients with dental anxiety (I^2^ = 32.7%, WMD (95% CI): −7.64(−9.43, −5.85), *p* < 0.0001). (Figure 7 and Supplementary File S3).

#### 3.2.8. Cooperation Rate

A total of 2 articles were included [17,20], including 188 patients. The results of the meta-analysis showed that music therapy significantly increased the cooperation rate of patients with dental anxiety (I^2^ = 0%, OR (95%CI): 3.03 (1.24, 7.40), *p* = 0.02, Supplementary File S3).

#### 3.2.9. Sensitivity Analysis and Publication Bias

Deletion of any of the studies for each of the outcome indicators did not significantly change the study results, suggesting that the results of this study were stable. Publication bias was examined for each outcome indicator within this study, and both Egger and Begger tests showed no significant publication bias (*p* < 0.05). Funnel plot evaluation showed a symmetrical appearance, indicating a low probability of bias (Supplementary File S3).

## 4. Discussion

People’s awareness of general oral hygiene and oral health care has gradually increased over the past few years as a result of the growth of the social economy and the advancement of science and technology, and they are now looking for effective solutions to oral health issues while also putting forth demands for oral treatment comfort [35]. Due to incorrect dental operations they have had in the past or childhood “white coat shadows,” some people suffer with dental anxiety or even phobias [36]. This anxiety is a patient’s subjective experience, and it can vary greatly depending on the person’s physical and mental health [37]. The anxiety that patients display during treatment is one of the major obstacles to dental comfort, and modern dental clinicians and researchers are increasingly realizing this. Failure to address this anxiety will probably lead to resistance to conventional treatment options and negatively impact treatment outcomes. A cost-effective and effective solution must be found.

Numerous physiological impacts of music on the human body have been documented, including changes in blood pressure, heart rate, respiration, and different metabolic reactions [38]. The value of music has been described as “the richest human emotional, sensory-motor, and cognitive experience”. Similar to dental anxiety, the effects of music are highly individualized, modulating cognition and mood by acting directly on the senses, reducing unpleasant sensations, enhancing mood, and boosting comfort and relaxation [39]. These effects are based on the patient’s past experiences and current state of mind. Therefore, it would appear that music is an allopathic treatment for dental anxiety. On this foundation, we conducted our investigation, which produced promising results. Patients in the test group who received an adjunctive music intervention significantly reduced their heart rate, blood pressure, pain perception, and anxiety compared to the conventional dental conventional treatment modality, and their dental treatment outcomes improved as a result. This finding is in line with the findings of a study by Bradt et al. [40], who found that using music in treatment may encourage relaxation and help to lower anxiety and stress levels. Further, the lack of fully consistent changes in outcome indicators across study groups in this study indicates that music therapy is not a traditional medication to explicitly reduce symptoms, but rather an embedded intervention that is part of the overall treatment, the effects of which are influenced by a number of factors. The implementation of music-assisted interventions should thoroughly assess the patient’s background information and current status to ensure that the interventions are effective. For the optimal clinical result, music-assisted therapies should be used in conjunction with a thorough evaluation of the patient’s history and current condition.

It should be noted that the results of the current study indicated no significant differences for the preoperative outcome indicators between the experimental and control groups, indicating consistent baseline levels, but significant heterogeneity between studies for postoperative heart rate, systolic and diastolic blood pressure, and pain scores. Due to this, we conducted a more thorough analysis to reduce within-group heterogeneity. To address potential statistical heterogeneity between studies, we performed a meta-analysis by random-effects model. We also took into account potential intraoperative heterogeneity due to various factors, such as different surgical stimulus responses in patients of different ages, inconsistent stimulus sizes due to various surgical difficulties, and different geographical regions The findings suggested that the aforementioned factors were not confounding factors because they did not significantly affect the results whether applied to different study populations, treatments, geographic regions, or years of publication. Sensitivity analysis also demonstrated that the study’s findings were consistent (Supplementary File S3). As a result, we think that the study’s findings are consistent and trustworthy and can serve as a guide for clinical care.

Additionally, formal music therapy refers to active interventions by a licensed music therapist or professional team (composing music, performing instruments, singing, and musical improvisation) or passive therapies (music listening) [41]. However, at the moment, the primary method of music intervention in dental anxiety disorders is that patients wear headphones and listen to music; as a result, the effectiveness of music in treating symptoms in patients with dental anxiety disorders may be constrained. Additionally, there are currently no professional methods of music therapy before and during surgery. In the future, a psychotherapist or music therapist may participate in more formal music therapy, which could produce better results.

The article has some flaws, including: (1) some outcome indicators’ heterogeneity could not be completely eliminated by statistical techniques, and we think that the study’s design, the type of music intervention, and the outcome indicators’ evaluation criteria are the main causes of the heterogeneity; (2) the included studies are all in Chinese and English, and it is unknown whether there are population differences in the study findings; (3) despite the robustness of the findings, some of the findings are based on small studies and may be subject to publication bias, which should be treated with caution; (4) the original study’s limitations prevented this meta-analysis from doing a more in-depth subgroup analysis to examine the impact of the treatment plan, the subject’s background, etc., on the clinical outcomes of music intervention therapy. However, it is undeniable that our study is the first pooled meta-analysis published that examines the use of music therapies to help treat patients with dental anxiety disorders. The future of dentistry is focused on providing comfortable dental care, and our study will undoubtedly offer reliable data for clinical use.

In conclusion, it can be inferred from a thorough analysis of the 18 clinical randomized controlled trials that were included that music therapy can help patients with dental anxiety disorder receive normal dental care by lowering their blood pressure and heart rate during procedures, as well as by easing their pain and anxiety. It can also increase patient cooperation. To further validate the study’s findings and obtain more unbiased and trustworthy clinical evidence, additional multicenter, large-sample, high-quality randomized controlled trials are required due to the study’s limitations.

## Figures and Tables

**Table 1:** Inclusion and exclusion criteria.

**Inclusion criteria**
a. Type of data Publicly published clinical randomized controlled trial (RCT) in Chinese or English; b. Study subjects Patients who received conventional oral treatment (including root canal treatment, periodontal treatment, tooth extraction, orthodontic treatment or restorative treatment) with a clear diagnosis of dental anxiety disorder, regardless of race, age, and gender; c. Interventions Patients in the test group were treated with music intervention + conventional oral treatment, while patients in the control group were treated with conventional oral treatment only, and the rest of the two groups remained the same; d. Outcome indicators Heart rate, systolic blood pressure, diastolic blood pressure, pain score, Modified Dental Anxiety Scale (MDAS), Children’s Fear Survey Schedule—Dental Schedule (CFSS-DS), State Anxiety Inventory (S-AI), and cooperation rate.
**Exclusion criteria**
a. Subject patients receiving unconventional oral treatment for oral cancer, oral mucosal lesions, etc., such as surgical resection; b. Conference abstracts, reviews, systematic reviews, basic experimental studies, meta-analyses, letters, case reports, or public database analyses; c. Study subjects or interventions inconsistent with the principles of univariate and randomized control; d. Insufficient sample size of subjects (less than 5 cases); e. Duplicate published literature or duplicate reported cases.

**Table 2:** Characteristics of included studies.

No.	Study	Country	Population	Operation	Number of Patients	Age	Outcome
1	Jian Yu, 2022 [17]	China	Child	Children’s Oral Treatment	60	T: 5.65 ± 1.19 C: 5.83 ± 1.12	CFSS-DS; Cooperation Rate
2	Juan Wang, 2021 [18]	China	Adult	Tooth Extraction	60	NA	Pain Score; MDAS
3	Huijun Hu, 2020 [19]	China	Adult	Tooth Extraction	58	NA	Pain Score; MDAS
4	Yizhu Wang, 2018 [20]	China	Child	Children’s Oral Treatment	128	NA	HR; SBP; DBP; Cooperation Rate
5	Xinzhi Yue, 2013 [21]	China	Adult	Oral Treatment	98	T: 32.9 ± 7.9 C: 33.1 ± 8.3	HR; SBP; DBP; Pain Score
6	Ning Ruan, 2011 [22]	China	Adult	Tooth Extraction	80	NA	Pain Score; MDAS; S-AI
7	Yang Gao, 2006 [23]	China	NA	Root Canal Treatment	42	T: 31.7 ± 8.3 C: 30.7 ± 8.3	Pain Score; S-AI
8	Haiyun Lu, 2008 [24]	China	Adult	Oral Treatment	100	NA	HR; SBP; DBP
9	Cynthia Mejía-Rubalcava, 2015 [25]	Mexico	Adult	Oral Treatment	34	NA	HR; SBP; DBP
10	Divya Singh, 2014 [26]	India	Child	Tooth Extraction	60	NA	HR; SBP; DBP
11	Yu-Kyoung Kim, 2011 [27]	Korea	Adult	Tooth Extraction	219	T: 35.1 ± 15.6 C: 38.6 ± 12.9	HR; SBP; DBP
12	Claas Lahmann, 2008 [28]	Germany	Adult	Oral Treatment	58	T: 32.7 ± 12.2 C: 43.7 ± 13.0	S-AI
13	Hui-Ling Lai, 2008 [29]	China	Adult	Root Canal Treatment	44	NA	HR; SBP; DBP; S-AI
14	Natthatida Janthasila, 2022 [30]	Thailand	Child	Children’s Oral Treatment	65	T: 11.00 ± 0.83 C: 11.00 ± 0.88	HR; SBP; DBP; CFSS-DS
15	Uma B Dixit, 2020 [31]	India	Child	Children’s Oral Treatment	80	T: 5.23 ± 0.11 C: 5.25 ± 0.12	HR; SBP; DBP
16	Jeswin James, 2021 [32]	India	Child	Children’s Oral Treatment	100	NA	HR
17	Serge Kalongo Tshiswaka, 2020 [33]	Brazil	Child	Children’s Oral Treatment	40	NA	HR; Pain Score
18	Ob-Om Buranavichetkul, 2021 [34]	Thailand	Adult	Periodontal Surgery	60	T: 48.03 ± 14.02 C: 52.53 ± 12.18	HR; SBP; DBP

Note: NA: not available; T: test group; C: control group; MDAS: Modified Dental Anxiety Scale; CFSS-DS: Children’s Fear Survey Schedule—Dental Schedule; S-AI: State Anxiety Inventory; HR: heart rate; SBP: systolic blood pressure; DBP: diastolic blood pressure.

## Data Availability

The data presented in this study are available in the main article and Supplementary Files.

## Supplementary Materials

The following supporting information can be downloaded at: https://www.mdpi.com/article/10.3390/medicina59020209/s1, Supplementary File S1: Keywords and search results; Supplementary File S2: Details of the included studies; Supplementary File S3: More analysis results; Supplementary File S4: Results of meta-regression analysis; Supplementary File S5: PICOs of the study and the consistency test of the investigators.
