# Supplementary material for: Efficacy of Music Intervention for Dental Anxiety Disorders: A Systematic Review and Meta-Analysis
Source: Medicina (Kaunas). 2023 Jan 20;59(2):209. doi: 10.3390/medicina59020209 (PMC9961880; doi:10.3390/medicina59020209)
Supplement: Supplementary file 1 [file medicina-59-00209-s001.zip › Supplementary file S1.pdf]

Supplementary file S1: Keywords and search result

Database or website	Strategy of searching	Latest retrieval time	Number of records
**Wan fang**	(oral OR dental) AND music	24 October, 2022	269
**CNKI**	(oral + dental) * music in Subject	24 October, 2022	346
**VIP**	Abstract = (oral OR dental) AND Abstract = music	24 October, 2022	184
**PubMed**	(oral OR dental) AND music in All Filed	24 October, 2022	980
**Web of Science**	((TS=oral) OR TS=(dental)) AND TS=(music)	24 October, 2022	1101
**Science Direct**	(oral OR dental) AND music in Title, abstract, keywords	24 October, 2022	104
**Cochrane Library**	(oral OR dental) AND music in Title Abstract Keyword	24 October, 2022	202
**Clinical tral.gov**	music | (oral OR dental)	24 October, 2022	28
**Chinese Clinical Trial Registry**	Music in Title	24 October, 2022	92
**Scopus**	(TITLE-ABS-KEY (oral) OR TITLE-ABS-KEY (dental) AND TITLE-ABS-KEY (music))	23 December, 2022	1656
**CINAHL**	AB oral OR AB dental AND AB music AND randomized controlled trials	23 December, 2022	126415
**References of included studies**	None	29 October, 2022	0

Note. CNKI: China National Knowledge Infrastructure; VIP: China Science and Technology Journal Database.
