# Supplementary material for: Efficacy of Music Intervention for Dental Anxiety Disorders: A Systematic Review and Meta-Analysis
Source: Medicina (Kaunas). 2023 Jan 20;59(2):209. doi: 10.3390/medicina59020209 (PMC9961880; doi:10.3390/medicina59020209)
Supplement: Supplementary file 1 [file medicina-59-00209-s001.zip › Supplementary file S2.pdf]

	Study	Group	Number	Male	Female	Age	HR Change	Preoperative HR	Postoperative HR	SBP Change	Preoperative SBP	Postoperative SBP	DBP Change	Preoperative DBP	Postoperative DBP	Scale Scoring Method 1	Change	Preoperative Score	Postoperative Score	Scale Scoring Method 2	Change	Preoperative Score	Postoperative Score	Scale Scoring Method 3	Change	Preoperative Score	Postoperative Score	Scale Scoring Method 4	Change	Preoperative Score	Postoperative Score
1	Jian Yu 2022	T	30	15	15	5.65±1.19	NA	NA	NA	NA	NA	NA	NA	NA	NA	CFSS-DS	-14.4±5.60	43.13±6.10	28.73±4.92	cooperation	NA	26/30	26/30	NA	NA	NA	NA	NA	NA	NA	NA
		C	30	16	14	5.83±1.12	NA	NA	NA	NA	NA	NA	NA	NA	NA		-5.37±6.07	42.27±5.84	36.90±6.28	rate	NA	18/30	18/30		NA	NA	NA		NA	NA	NA
2	Juan Wang 2021	T	30	6	24	NA	NA	NA	NA	NA	NA	NA	NA	NA	NA	MDAS	-3.40±3.42	16.23±1.61	12.83±3.43	VAS	-1.78±2.39	3.02±2.20	1.23±1.30	NA	NA	NA	NA	NA	NA	NA	NA
		C	30	7	23	NA	NA	NA	NA	NA	NA	NA	NA	NA	NA		-0.07±2.43	15.87±1.38	15.80±2.75		0.21±1.02	2.91±1.28	3.12±1.44		NA	NA	NA		NA	NA	NA
3	Huijun Hu 2020	T	30	NA	NA	NA	NA	NA	NA	NA	NA	NA	NA	NA	NA	MDAS	-2.98±2.10	14.23±2.35	11.25±1.69	VAS	-3.48±1.94	6.12±2.18	2.64±1.54	NA	NA	NA	NA	NA	NA	NA	NA
		C	28	NA	NA	NA	NA	NA	NA	NA	NA	NA	NA	NA	NA		-0.08±2.17	14.34±2.43	14.26±1.74		-0.2±1.95	6.18±2.14	5.98±1.67		NA	NA	NA		NA	NA	NA
4	Yizhu Wang 2018	T	64	NA	NA	NA	1.44±15.92	108±15.8	109.44±16.04	-5±10.10	104.89±11.63	99.89±6.58	0.44±8.77	60.11±9.25	60.55±8.19	NA	NA	NA	NA	cooperation	NA	NA	60/64	NA	NA	NA	NA	NA	NA	NA	NA
		C	64	NA	NA	NA	18.38±40.62	95.45±34.88	113.83±44.59	4.83±11.37	106.11±10.62	110.94±11.99	4.33±13.48	66.06±10.68	70.39±15.14		NA	NA	NA	rate	NA	NA	56/64		NA	NA	NA		NA	NA	NA
5	Xinzhi Yue 2013	T	49	23	26	32.9±7.9	2.1±6.56	76.1±6.0	78.2±7.0	1.3±5.27	120.7±5.5	122±5	-2.1±5.1	75.1±5.2	73±5	NA	NA	NA	NA	NRS（VAS）	NA	NA	2.5±0.6	NA	NA	NA	NA	NA	NA	NA	NA
		C	49	25	24	33.1±8.3	4.1±6.24	77.2±5.8	81.3±6.6	4.1±5.35	119.6±5.3	123.7±5.4	-0.8±5.25	78.1±5.3	77.3±5.2		NA	NA	NA		NA	NA	3.0±0.7		NA	NA	NA		NA	NA	NA
6	Ning Ruan 2011	T	40	12	28	NA	NA	NA	NA	NA	NA	NA	NA	NA	NA	MDAS	-2.74±2.74	14.20±2.34	11.46±3.02	S-AI	-10.41±5.56	55.18±5.03	44.77±5.97	HAMA	-2.37±2.60	7.52±2.66	5.15±2.53	VAS	-1.8±2.25	7.20±1.83	5.40±2.51
		C	40	14	26	NA	NA	NA	NA	NA	NA	NA	NA	NA	NA		-0.13±3.70	14.37±3.10	14.24±4.10		-0.25±8.39	54.62±4.21	54.37±9.66		0.38±3.1	7.23±2.67	7.61±3.40		-0.36±2.10	6.63±2.18	6.27±2.00
7	Yang Gao 2006	T	21	11	10	31.7±8.3	NA	NA	NA	NA	NA	NA	NA	NA	NA	S-AI	-22.29±3.70	56.38±7.72	34.10±6.48	VAS	-5.43±1.75	7.98±1.67	2.55±1.32	NA	NA	NA	NA	NA	NA	NA	NA
		C	21	10	11	30.7±8.3	NA	NA	NA	NA	NA	NA	NA	NA	NA		-9.24±4.49	52.95±8.89	43.71±7.83		-3.88±1.56	7.71±1.67	3.88±1.56		NA	NA	NA		NA	NA	NA
8	Haiyun Lu 2008	T	50	NA	NA	NA	1.4±5.11	70.7±5.3	72.1±4.9	4.3±6.22	103.8±4.5	108.1±7.1	4±4.69	63.4±5.4	67.4±3.0	DAS	0.19±1.73	14.1±1.1	14.29±2.00	NA	NA	NA	NA	NA	NA	NA	NA	NA	NA	NA	NA
		C	50	NA	NA	NA	7.5±4.8	70.2±5.5	77.7±2.9	8.4±5.92	102.2±6.8	110.6±4.0	7.6±3.57	62.7±3.9	70.3±3.1		0.45±1.73	14.4±1.1	14.85±2.00		NA	NA	NA		NA	NA	NA		NA	NA	NA
9	Cynthia Mejía-Rubalcava 2015	T	17	6	11	35.1±15.6	-7.7±9.1	78.8±10.5	71.1±5.6	-9.2±8.41	131.7±9.1	122.5±7.5	-11.6±8.35	86.5±9.0	74.9 ± 7.5	NA	NA	NA	NA	NA	NA	NA	NA	NA	NA	NA	NA	NA	NA	NA	NA
		C	17	5	12	38.6±12.9	3.4±6.36	84.5±6.1	87.9 ± 6.6	3.3±5.57	131.6±4.6	134.9±6.2	1.6±5.81	87.2±4.7	88.8±6.5		NA	NA	NA		NA	NA	NA		NA	NA	NA		NA	NA	NA
10	Divya Singh 2014	T	30	NA	NA	NA	-13.7±12.8	100.5±15.4	87.1±12.1	-3.4±5.7	122.3±12.5	118.8±12.1	-1.5±7.1	79.7±12.6	78.2±12.5	Venham	-3.00±2.15	4.83 ± 2.20	1.83 ± 1.68	NA	NA	NA	NA	NA	NA	NA	NA	NA	NA	NA	NA
		C	30	NA	NA	NA	5.0±17.4	102.4±15.8	107.4±19.8	3.8±6.7	124.0±12.2	127.8 ± 9.5	2.2±7.7	81.2±10.8	83.4±8.0	(vpt)	1.90±1.32	4.43 ± 1.70	6.33 ± 1.45		NA	NA	NA		NA	NA	NA		NA	NA	NA
11	Yu-Kyoung Kim 2011	T	106	59	47	NA	-15.76±8.16	88.30 ± 9.20	72.54±6.37	-1.88±13.55	120.49±14.49	118.61±12.36	-3.99±6.82	78.62 ± 7.64	74.63 ± 5.46	DAS	-0.3±3.17	13.42 ± 3.09	13.12 ± 3.24	NA	NA	NA	NA	NA	NA	NA	NA	NA	NA	NA	NA
		C	113	63	50	NA	-14.65±12.43	90.69±10.24	76.04±13.83	-5.34±13.73	123.26±15.55	117.92±10.45	-1.01±10.59	78.39 ± 9.85	77.38 ± 11.20		0.36±3.00	13.15 ± 2.87	13.51 ± 3.11		NA	NA	NA		NA	NA	NA		NA	NA	NA
12	Claas Lahmann 2008	T	28	11	17	32.7±12.2	NA	NA	NA	NA	NA	NA	NA	NA	NA	S-AI	-4.4±4.6	41.3±9.6	36.8±9.8	NA	NA	NA	NA	NA	NA	NA	NA	NA	NA	NA	NA
		C	30	18	12	43.7±13.0	NA	NA	NA	NA	NA	NA	NA	NA	NA		-1.4±4.4	41.9±11.5	40.5±11.2		NA	NA	NA		NA	NA	NA		NA	NA	NA
13	Hui-Ling Lai 2008	T	22	14	8	NA	-5.68±6.21	77.23± 6.65	71.55± 5.65	-8.23±11.48	135.73±11.83	127.50±11.10	0.77±8.82	84.14 ±7.86	84.91 ± 9.54	S-AI	-17.84±4.54	50.64±5.10	32.80±3.61	NA	NA	NA	NA	NA	NA	NA	NA	NA	NA	NA	NA

		C	22	7	15	NA	-4.59±7.41	81.41± 7.45	76.82±7.36	9.45±13.88	136.00±12.78	145.45±14.76	2.18±8.18	78.32 ± 8.58	80.50 ± 7.71		-10.18±5.33	49.73±5.49	39.55±5.16		NA	NA	NA		NA	NA	NA		NA	NA	NA
14	Natthatida Janthasila 2022	T	33	13	20	11.00± 0.83	'-1.0±1.5	83.6±10.8	82.6± 9.9	'-3.0±1.2	104.2±12.9	101.2± 11.3	-1.6±1.1	68.9 ± 8.7	67.3± 7.9	CFSS-DS	-12.9±1.3	35.3 ± 5.1	22.4± 7.3	NA	NA	NA	NA	NA	NA	NA	NA	NA	NA	NA	NA
		C	32	19	13	11.00± 0.88	6.5±1.4	85.0 ± 6.2	91.5± 9.1	1.8±1.4	104.7± 13.0	106.5± 11.7	1.5±1.0	67.7±10.4	69.2± 9.8		'-1.6±1.4	35.2 ± 7.4	33.6± 10.8		NA	NA	NA		NA	NA	NA		NA	NA	NA
15	Uma B Dixit 2020	T	40	22	18	5.23 ± 0.11	-2.6±13.55	105.5±13.6	102.9±13.5	1±6.62	109±6	110± 7.1	NA	NA	NA	NA	NA	NA	NA	NA	NA	NA	NA	NA	NA	NA	NA	NA	NA	NA	NA
		C	40	26	14	5.25 ± 0.12	0.3±12.22	108 ±12.6	108.3±11.8	-0.1±8.01	112.3±7.4	112.2± 8.5	NA	NA	NA		NA	NA	NA		NA	NA	NA		NA	NA	NA		NA	NA	NA
16	Jeswin James	T	50	NA	NA	NA	-9.48±11.97	100.5±13.18	91.02 ± 10.2	NA	NA	NA	NA	NA	NA	NA	NA	NA	NA	NA	NA	NA	NA	NA	NA	NA	NA	NA	NA	NA	NA
		C	50	NA	NA	NA	4.8±12.04	98.4 ± 12.74	103.2±11.2	NA	NA	NA	NA	NA	NA		NA	NA	NA		NA	NA	NA		NA	NA	NA		NA	NA	NA
17	Serge Kalongo TSHISWAKA	T	20	14	6	NA	-8.8±15.31	93.80±14.85	85.00±15.73	NA	NA	NA	NA	NA	NA	VAS	-0.6±1.59	1.60±1.66	1.00±1.52	NA	NA	NA	NA	NA	NA	NA	NA	NA	NA	NA	NA
	2021	C	20	9	11	NA	-4.2±14.95	91.35±14.36	87.15±15.48	NA	NA	NA	NA	NA	NA		-1±3.02	2.10±3.40	1.10±2.38		NA	NA	NA		NA	NA	NA		NA	NA	NA
18	Ob-Om Buranavichetkul 2021	T	30	17	13	48.03±14.02	-8.4±12.31	76.60±12.45	68.20±12.17	1.37±23.08	120.93±13.81	122.30±26.65	4.7±9.68	68.87±9.40	73.57±9.94	VPT	-0.34±0.88	2.37±0.86	2.03±0.89	NA	NA	NA	NA	NA	NA	NA	NA	NA	NA	NA	NA
		C	30	20	10	52.53±12.18	-9.04±14.71	77.57±11.95	68.53±16.43	3.83±16.30	123.47±16.03	127.30±16.56	6.2±14.37	70.30±16.55	76.50±9.25		-0.8±0.91	2.47±1.01	1.67±0.76		NA	NA	NA		NA	NA	NA		NA	NA	NA
