# Supplementary material for: Efficacy of Music Intervention for Dental Anxiety Disorders: A Systematic Review and Meta-Analysis
Source: Medicina (Kaunas). 2023 Jan 20;59(2):209. doi: 10.3390/medicina59020209 (PMC9961880; doi:10.3390/medicina59020209)

1. Heart Rate (HR)

1.1 HR Change

Study or Subgroup	Experimental Mean	Experimental SD	Experimental Total	Control Mean	Control SD	Control Total	Weight	Std. Mean Difference IV, Fixed, 95% CI
Cynthia Mejía-Rubalcava 2015	-7.7	9.1	17	3.4	6.36	17	2.9%	-1.38 [-2.14, -0.62]
Divya Singh 2014	-13.7	12.8	30	5	17.4	30	5.4%	-1.21 [-1.76, -0.66]
Haiyun Lu 2008	1.4	5.11	50	7.5	4.8	50	9.1%	-1.22 [-1.65, -0.79]
Hui-Ling Lai 2008	-5.68	6.21	22	-4.59	7.41	22	4.8%	-0.16 [-0.75, 0.44]
Jeswin James 2021	-9.48	11.97	50	4.8	12.04	50	9.2%	-1.18 [-1.61, -0.75]
Natthatida Janthasila 2022	-1	1.5	33	6.5	1.4	32	1.6%	-5.10 [-6.13, -4.08]
Ob-Om Buranavichetkul 2021	-8.4	12.31	30	-9.04	14.71	30	6.5%	0.05 [-0.46, 0.55]
Serge Kalongo TSHISWAKA 2020	-8.8	15.31	20	-4.2	14.95	20	4.3%	-0.30 [-0.92, 0.33]
Uma B Dixit 2020	-2.6	13.55	40	0.3	12.22	40	8.6%	-0.22 [-0.66, 0.22]
Xinzhi Yue 2013	2.1	6.56	49	4.1	6.24	49	10.5%	-0.31 [-0.71, 0.09]
Yizhu Wang 2018	1.44	15.92	64	18.38	40.62	64	13.4%	-0.55 [-0.90, -0.19]
Yu-Kyoung Kim 2011	-15.76	8.16	106	-14.65	12.43	113	23.7%	-0.10 [-0.37, 0.16]
**Total (95% CI)**			**511**			**517**	**100.0%**	**-0.57 [-0.70, -0.44]**

Heterogeneity: $Chi^2 = 125.26$, df = 11 ($P < 0.00001$); $I^2 = 91\%$

Test for overall effect: $Z = 8.69$ ($P < 0.00001$)

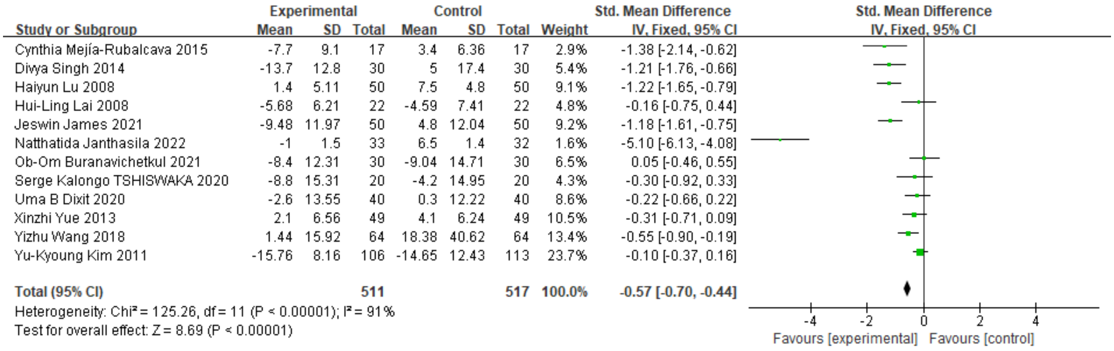

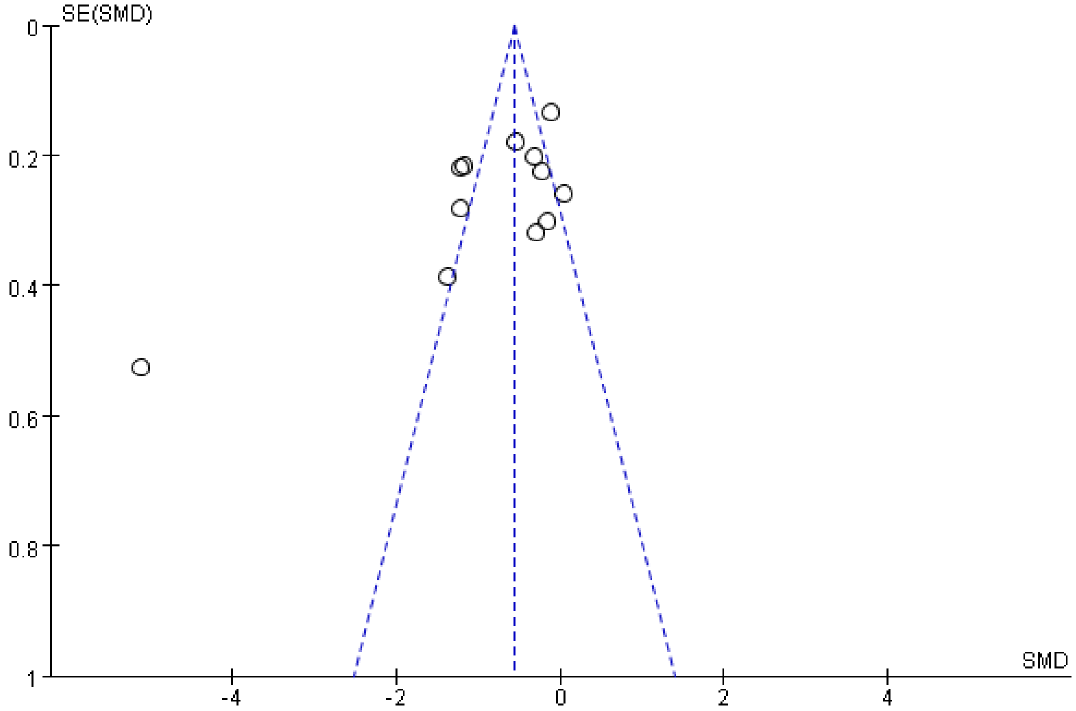

2. Systolic Blood Pressure (SBP)

2.1 Preoperative SBP

Study or Subgroup	Experimental Mean	Experimental SD	Experimental Total	Control Mean	Control SD	Control Total	Weight	Std. Mean Difference IV, Fixed, 95% CI
Cynthia Mejía-Rubalcava 2015	131.7	9.1	17	131.6	4.6	17	3.9%	0.01 [-0.66, 0.69]
Divya Singh 2014	122.3	12.5	30	124	12.2	30	6.8%	-0.14 [-0.64, 0.37]
Haiyun Lu 2008	103.8	4.5	50	102.2	6.8	50	11.2%	0.28 [-0.12, 0.67]
Hui-Ling Lai 2008	135.73	11.83	22	136	12.78	22	5.0%	-0.02 [-0.61, 0.57]
Natthatida Janthasila 2022	104.2	12.9	33	104.7	13	32	7.4%	-0.04 [-0.52, 0.45]
Ob-Om Buranavichetkul 2021	120.93	13.81	30	123.47	16.03	30	6.8%	-0.17 [-0.67, 0.34]
Uma B Dixit 2020	109	6	40	112.3	7.4	40	8.8%	-0.49 [-0.93, -0.04]
Xinzhi Yue 2013	120.7	5.5	49	119.6	5.3	49	11.0%	0.20 [-0.19, 0.60]
Yizhu Wang 2018	104.89	11.63	64	106.11	10.62	64	14.5%	-0.11 [-0.46, 0.24]
Yu-Kyoung Kim 2011	120.49	14.49	106	123.26	15.55	113	24.7%	-0.18 [-0.45, 0.08]
**Total (95% CI)**			**441**			**447**	**100.0%**	**-0.07 [-0.21, 0.06]**

Heterogeneity: $Chi^2 = 9.15$, df = 9 ($P = 0.42$); $I^2 = 2\%$

Test for overall effect: $Z = 1.11$ ($P = 0.27$)

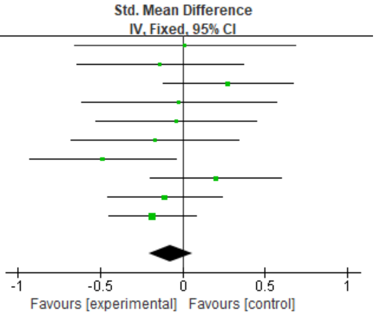

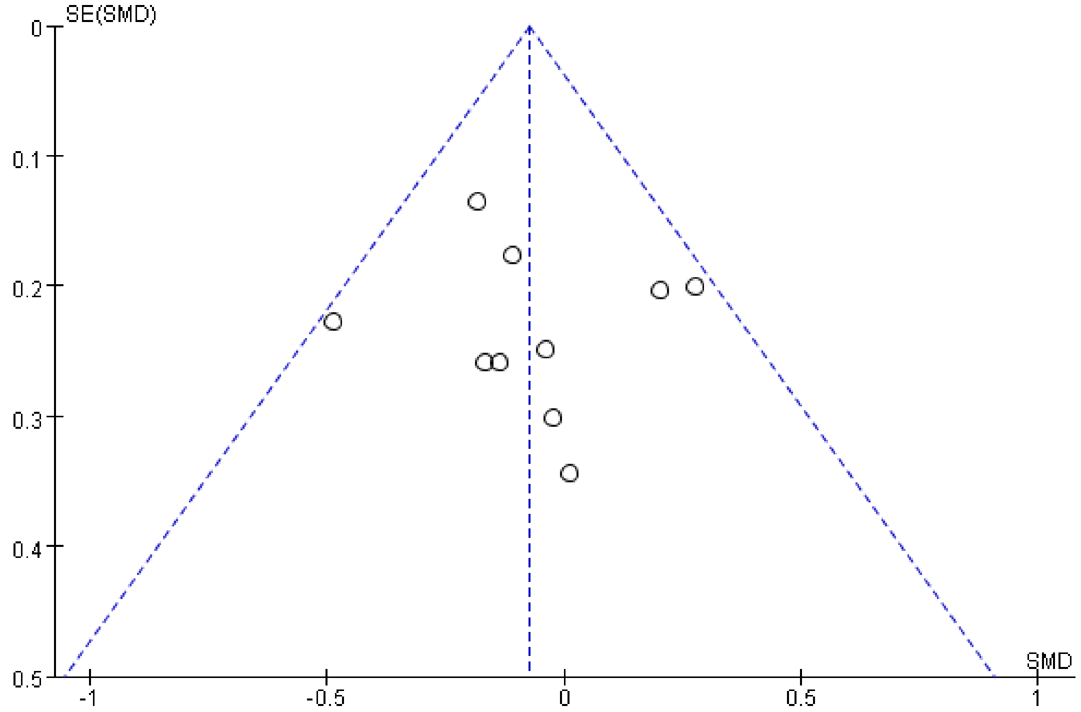

2.2 Postoperative SBP

Study or Subgroup	Experimental Mean	Experimental SD	Experimental Total	Control Mean	Control SD	Control Total	Weight	Std. Mean Difference IV, Fixed, 95% CI
Cynthia Mejía-Rubalcava 2015	122.5	7.5	17	134.9	6.2	17	2.8%	-1.76 [-2.57, -0.95]
Divya Singh 2014	118.8	12.1	30	127.8	9.5	30	6.6%	-0.82 [-1.34, -0.29]
Haiyun Lu 2008	108.1	7.1	50	110.6	4	50	11.6%	-0.43 [-0.83, -0.03]
Hui-Ling Lai 2008	127.5	11.1	22	145.45	14.76	22	4.2%	-1.35 [-2.01, -0.69]
Natthatida Janthasila 2022	101.2	11.3	33	106.5	11.7	32	7.5%	-0.46 [-0.95, 0.04]
Ob-Om Buranavichetkul 2021	122.3	26.65	30	127.3	16.56	30	7.1%	-0.22 [-0.73, 0.29]
Uma B Dixit 2020	110	7.1	40	112.2	8.5	40	9.4%	-0.28 [-0.72, 0.16]
Xinzhi Yue 2013	122	5	49	123.7	5.4	49	11.5%	-0.32 [-0.72, 0.07]
Yizhu Wang 2018	99.89	6.58	64	110.94	11.99	64	13.1%	-1.14 [-1.51, -0.76]
Yu-Kyoung Kim 2011	118.61	12.36	106	117.92	10.45	113	26.1%	0.06 [-0.20, 0.33]
**Total (95% CI)**			**441**			**447**	**100.0%**	**-0.46 [-0.59, -0.32]**

Heterogeneity: Chi² = 47.99, df = 9 (P < 0.00001); I² = 81%
Test for overall effect: Z = 6.61 (P < 0.00001)

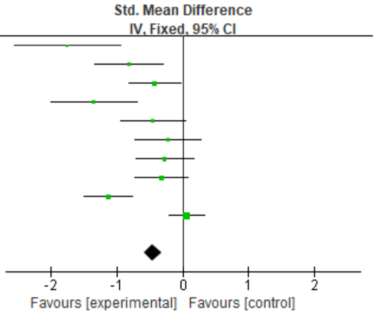

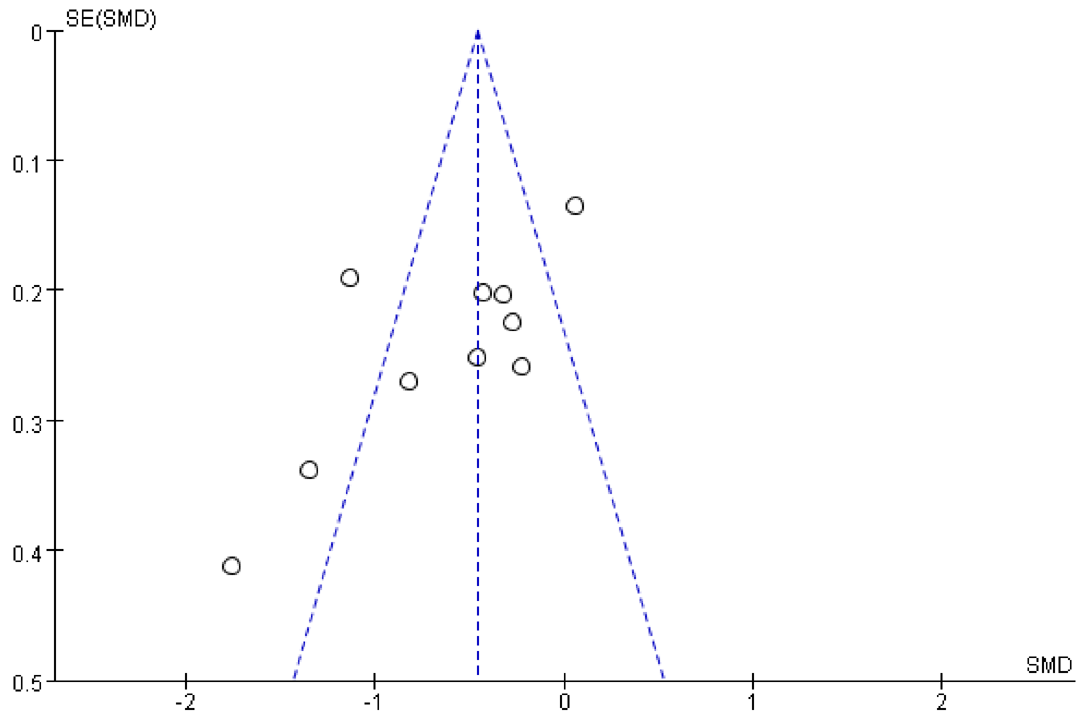

2.3 SBP Change

Study or Subgroup	Experimental Mean	Experimental SD	Experimental Total	Control Mean	Control SD	Control Total	Weight	Std. Mean Difference IV, Fixed, 95% CI
Cynthia Mejía-Rubalcava 2015	-9.2	8.41	17	3.3	5.57	17	3.0%	-1.71 [-2.51, -0.91]
Divya Singh 2014	-3.4	5.7	30	3.8	6.7	30	6.4%	-1.14 [-1.69, -0.59]
Haiyun Lu 2008	4.3	6.22	50	8.4	5.92	50	11.9%	-0.67 [-1.07, -0.27]
Hui-Ling Lai 2008	-8.23	11.48	22	9.45	13.88	22	4.4%	-1.36 [-2.03, -0.70]
Natthatida Janthasila 2022	-3	1.2	33	1.8	1.4	32	3.0%	-3.64 [-4.45, -2.83]
Ob-Om Buranavichetkul 2021	1.37	23.08	30	3.83	16.3	30	7.5%	-0.12 [-0.63, 0.39]
Uma B Dixit 2020	1	6.62	40	-0.1	8.01	40	10.0%	0.15 [-0.29, 0.59]
Xinzhi Yue 2013	1.3	5.27	49	4.1	5.35	49	11.9%	-0.52 [-0.93, -0.12]
Yizhu Wang 2018	-5	10.1	64	4.83	11.37	64	14.5%	-0.91 [-1.27, -0.54]
Yu-Kyoung Kim 2011	-1.88	13.55	106	-5.34	13.73	113	27.3%	0.25 [-0.01, 0.52]
**Total (95% CI)**			**441**			**447**	**100.0%**	**-0.49 [-0.63, -0.35]**

Heterogeneity: Chi² = 125.41, df = 9 (P < 0.00001); I² = 93%
Test for overall effect: Z = 6.94 (P < 0.00001)

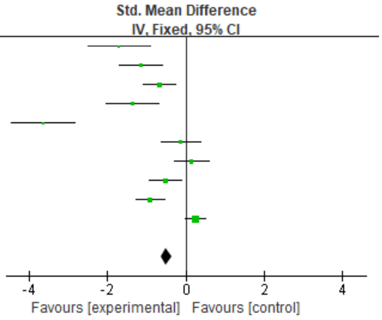

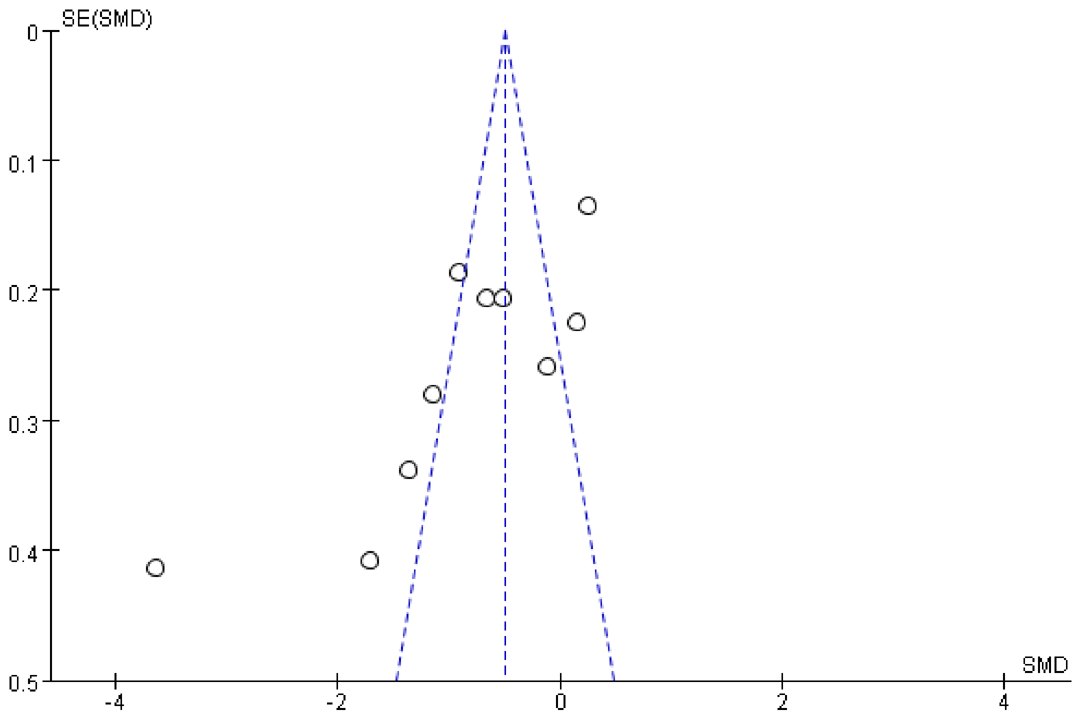

3. Diastolic Blood Pressure (DBP)

3.1 Preoperative DBP

Study or Subgroup	Experimental Mean	Experimental SD	Experimental Total	Control Mean	Control SD	Control Total	Weight	Std. Mean Difference IV, Fixed, 95% CI
Cynthia Mejía-Rubalcava 2015	86.5	9	17	87.2	4.7	17	4.3%	-0.10 [-0.77, 0.58]
Divya Singh 2014	79.7	12.6	30	81.2	10.8	30	7.5%	-0.13 [-0.63, 0.38]
Haiyun Lu 2008	63.4	5.4	50	62.7	3.9	50	12.5%	0.15 [-0.25, 0.54]
Hui-Ling Lai 2008	84.14	7.86	22	78.32	8.58	22	5.2%	0.69 [0.08, 1.30]
Natthatida Janthasila 2022	68.9	8.7	33	67.7	10.4	32	8.2%	0.12 [-0.36, 0.61]
Ob-Om Buranavichetkul 2021	68.87	9.4	30	70.3	16.55	30	7.5%	-0.10 [-0.61, 0.40]
Xinzhi Yue 2013	75.1	5.2	49	78.1	5.3	49	11.8%	-0.57 [-0.97, -0.16]
Yizhu Wang 2018	60.11	9.25	64	66.06	10.68	64	15.4%	-0.59 [-0.95, -0.24]
Yu-Kyoung Kim 2011	78.62	7.64	106	78.39	9.85	113	27.5%	0.03 [-0.24, 0.29]
**Total (95% CI)**			**401**			**407**	**100.0%**	**-0.11 [-0.25, 0.03]**

Heterogeneity: $Chi^2 = 22.26$, df = 8 (P = 0.004); $I^2 = 64\%$

Test for overall effect: Z = 1.52 (P = 0.13)

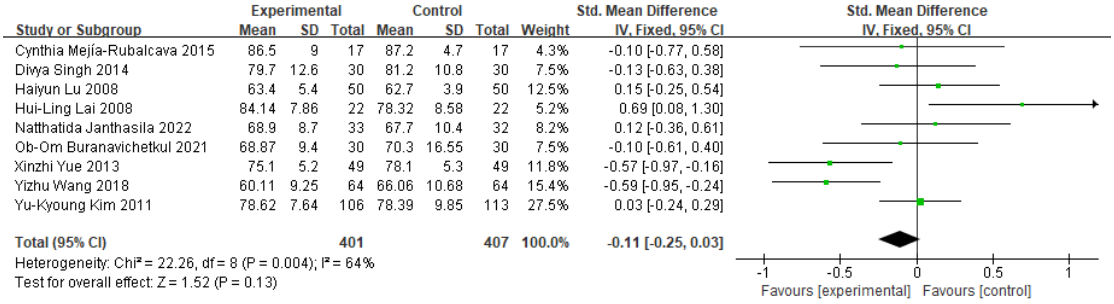

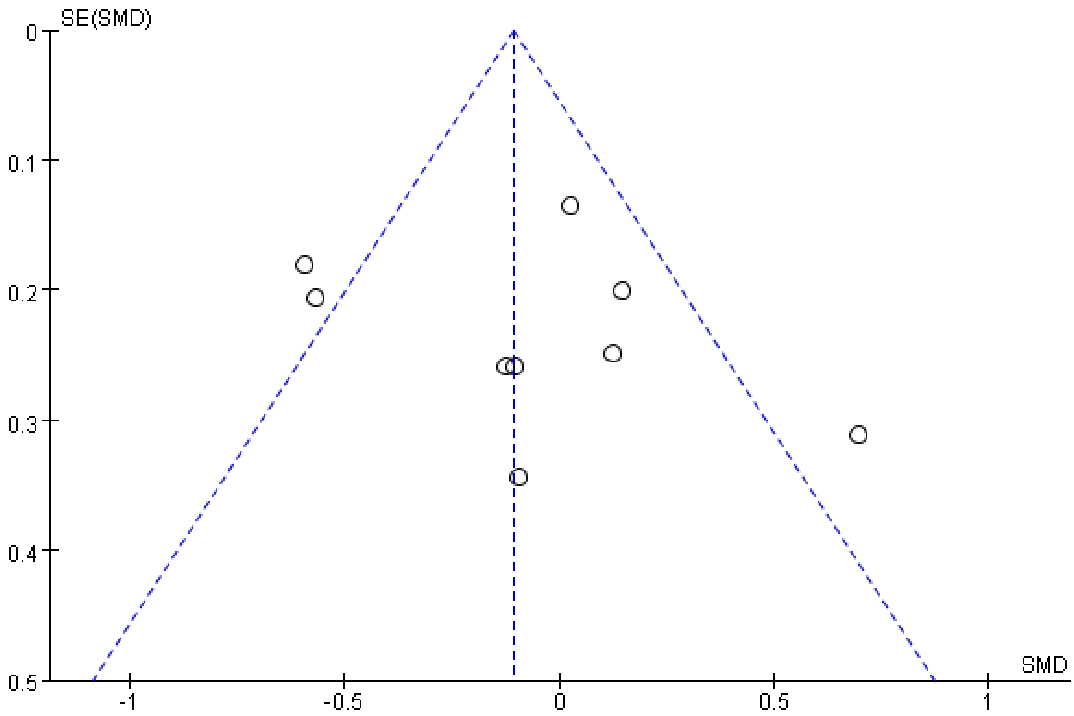

3.2 Postoperative DBP

	Experimental			Control				Std. Mean Difference
Study or Subgroup	Mean	SD	Total	Mean	SD	Total	Weight	IV, Fixed, 95% CI
Cynthia Mejía-Rubalcava 2015	74.9	7.5	17	88.8	6.5	17	2.9%	-1.93 [-2.77, -1.10]
Divya Singh 2014	78.2	12.5	30	83.4	8	30	7.6%	-0.49 [-1.00, 0.03]
Haiyun Lu 2008	67.4	3	50	70.3	3.1	50	11.8%	-0.94 [-1.36, -0.53]
Hui-Ling Lai 2008	84.91	9.54	22	80.5	7.71	22	5.6%	0.50 [-0.10, 1.10]
Natthatida Janthasila 2022	67.3	7.9	33	69.2	9.8	32	8.5%	-0.21 [-0.70, 0.28]
Ob-Om Buranavichetkul 2021	73.57	9.94	30	76.5	9.25	30	7.8%	-0.30 [-0.81, 0.21]
Xinzhi Yue 2013	73	5	49	77.3	5.2	49	11.8%	-0.84 [-1.25, -0.42]
Yizhu Wang 2018	60.55	8.19	64	70.39	15.14	64	15.5%	-0.80 [-1.16, -0.44]
Yu-Kyoung Kim 2011	74.63	5.46	106	77.38	11.2	113	28.4%	-0.31 [-0.57, -0.04]
**Total (95% CI)**			**401**			**407**	**100.0%**	**-0.53 [-0.67, -0.39]**

Heterogeneity: Chi² = 35.48, df = 8 (P < 0.0001); I² = 77%
Test for overall effect: Z = 7.30 (P < 0.00001)

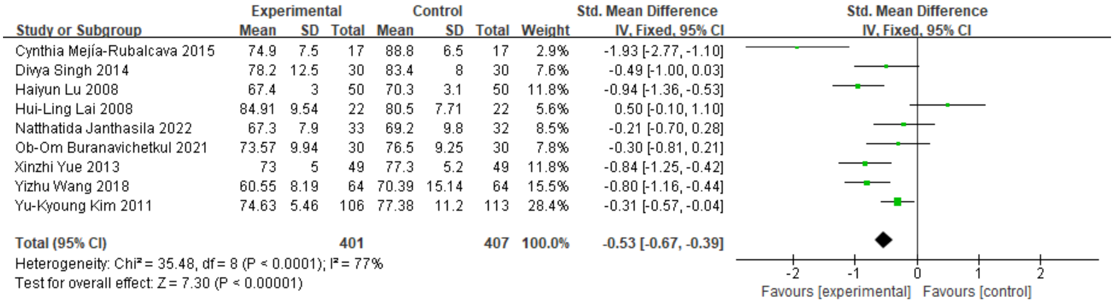

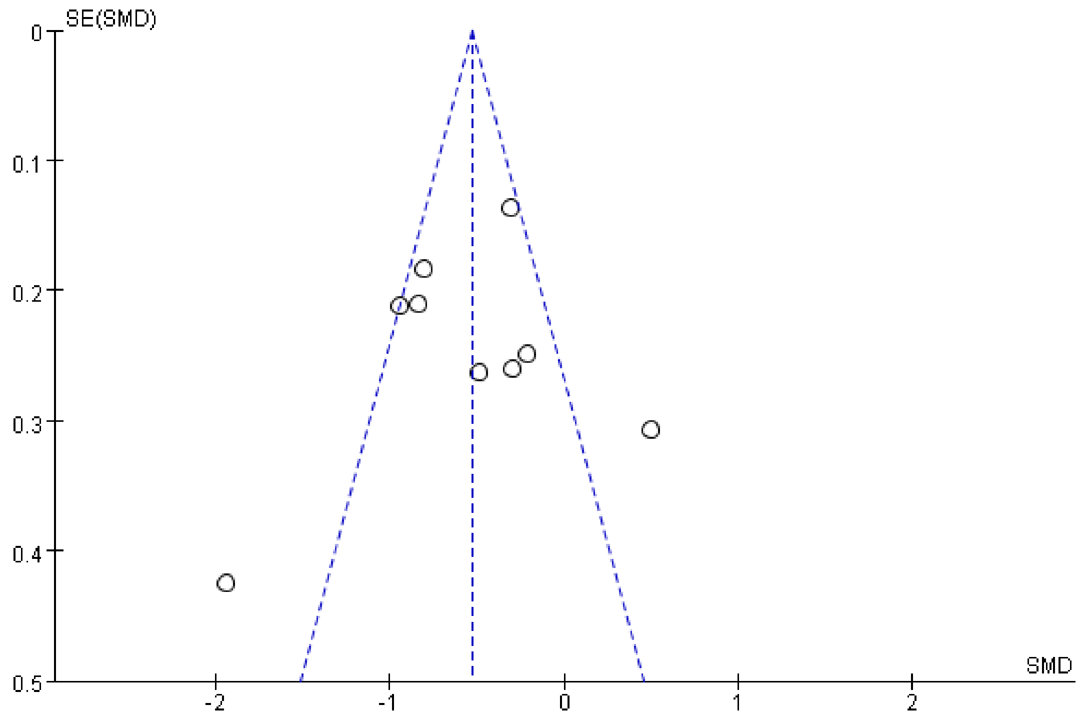

3.3 DBP Change

Study or Subgroup	Experimental Mean	Experimental SD	Experimental Total	Control Mean	Control SD	Control Total	Weight	Std. Mean Difference IV, Fixed, 95% CI
Cynthia Mejía-Rubalcava 2015	-11.6	8.35	17	1.6	5.81	17	3.1%	-1.79 [-2.60, -0.98]
Divya Singh 2014	-1.5	7.1	30	2.2	5.81	30	7.7%	-0.56 [-1.08, -0.05]
Haiyun Lu 2008	4	4.69	50	7.6	3.57	50	12.2%	-0.86 [-1.27, -0.45]
Hui-Ling Lai 2008	0.77	8.82	22	2.18	8.18	22	5.9%	-0.16 [-0.75, 0.43]
Natthatida Janthasila 2022	-1.6	1.1	33	1.5	1	32	4.1%	-2.91 [-3.62, -2.20]
Ob-Om Buranavichetkul 2021	4.7	9.68	30	6.2	14.37	30	8.0%	-0.12 [-0.63, 0.39]
Xinzhi Yue 2013	-2.1	5.1	49	-0.8	5.25	49	13.0%	-0.25 [-0.65, 0.15]
Yizhu Wang 2018	0.44	8.77	64	4.33	13.48	64	16.9%	-0.34 [-0.69, 0.01]
Yu-Kyoung Kim 2011	-3.99	6.82	106	-1.01	10.59	113	28.9%	-0.33 [-0.60, -0.06]
**Total (95% CI)**			**401**			**407**	**100.0%**	**-0.53 [-0.67, -0.39]**

Heterogeneity: Chi² = 64.24, df = 8 (P < 0.00001); I² = 88%
Test for overall effect: Z = 7.22 (P < 0.00001)

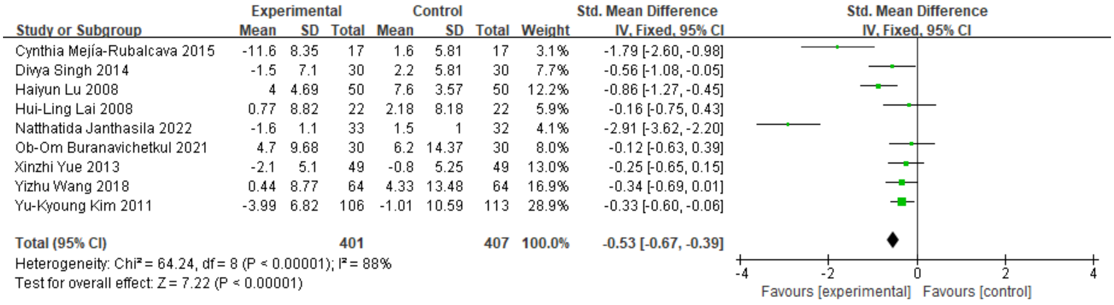

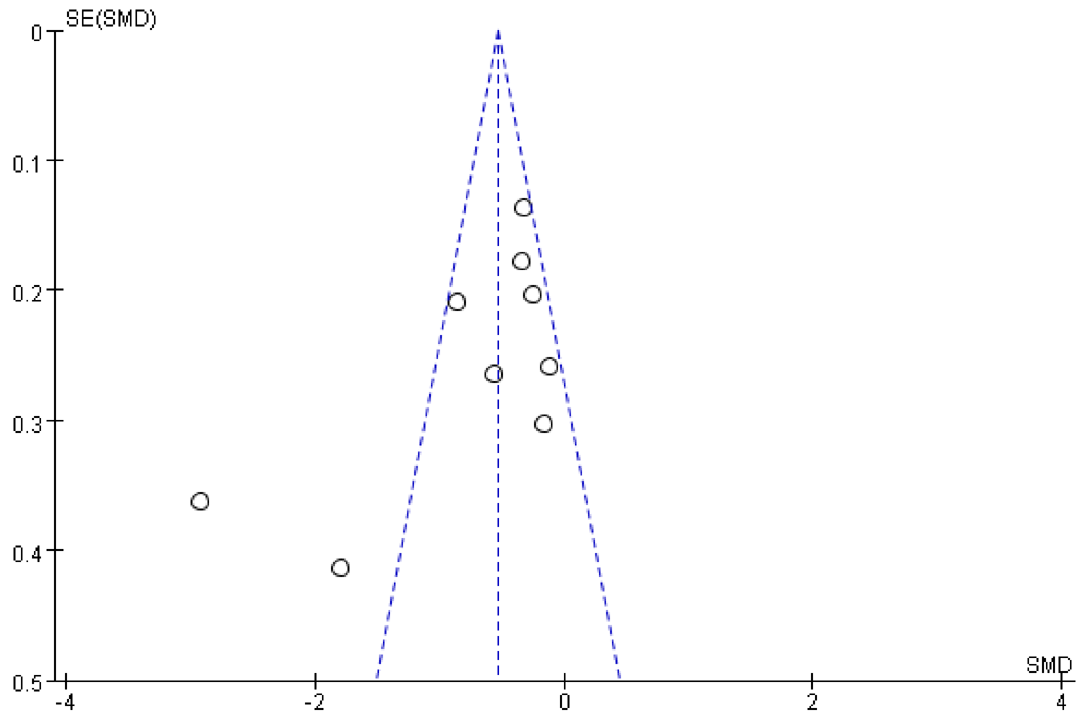

4. Cooperation Rate

4.1 Cooperation Rate Change

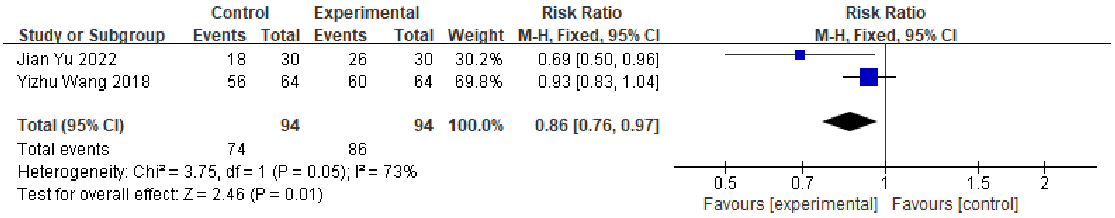

Study or Subgroup	Control Events	Control Total	Experimental Events	Experimental Total	Weight	Risk Ratio M-H, Fixed, 95% CI
Jian Yu 2022	18	30	26	30	30.2%	0.69 [0.50, 0.96]
Yizhu Wang 2018	56	64	60	64	69.8%	0.93 [0.83, 1.04]
**Total (95% CI)**		**94**		**94**	**100.0%**	**0.86 [0.76, 0.97]**
Total events	74		86			

Heterogeneity: Chi² = 3.75, df = 1 (P = 0.05); I² = 73%

Test for overall effect: Z = 2.46 (P = 0.01)

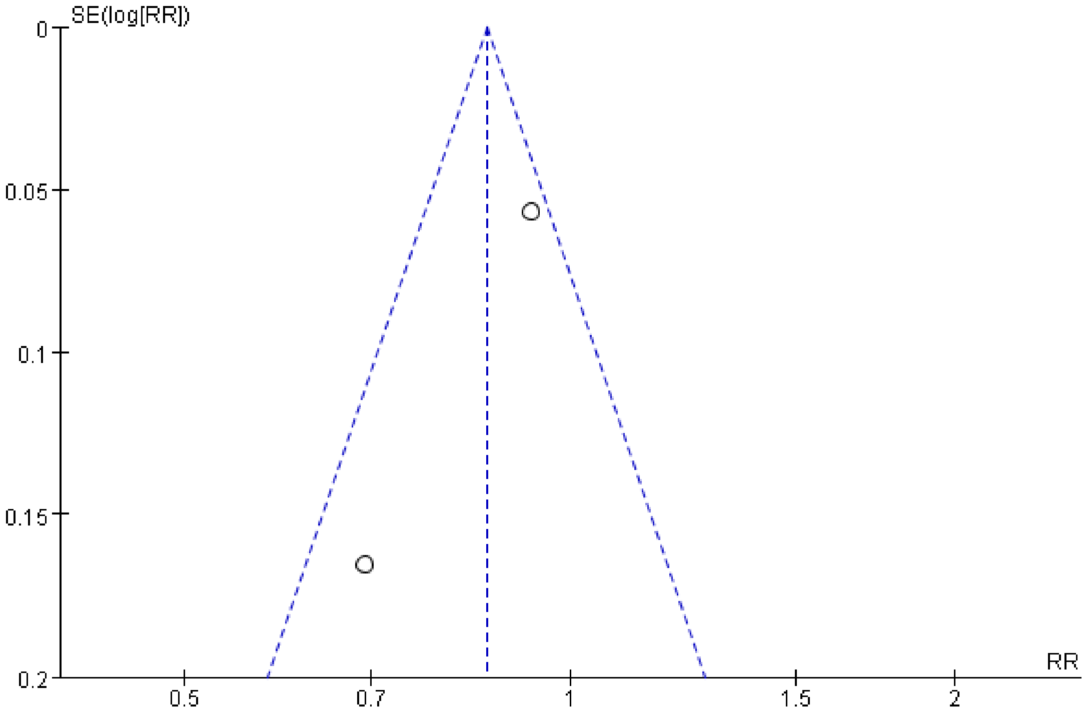

5. CFSS-DS

5.1 Preoperative CFSS-DS

Study or Subgroup	Experimental Mean	Experimental SD	Experimental Total	Control Mean	Control SD	Control Total	Weight	Std. Mean Difference IV, Fixed, 95% CI
Jian Yu 2022	43.13	6.1	30	42.27	5.84	30	47.9%	0.14 [-0.36, 0.65]
Natthatida Janthasila 2022	35.3	5.1	33	35.2	7.4	32	52.1%	0.02 [-0.47, 0.50]
**Total (95% CI)**			**63**			**62**	**100.0%**	**0.08 [-0.27, 0.43]**

Heterogeneity: Chi² = 0.12, df = 1 (P = 0.72); I² = 0%
Test for overall effect: Z = 0.43 (P = 0.67)

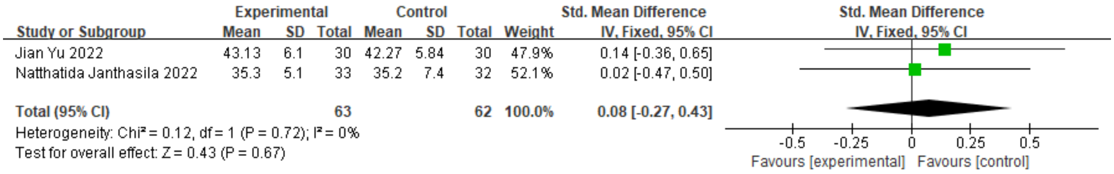

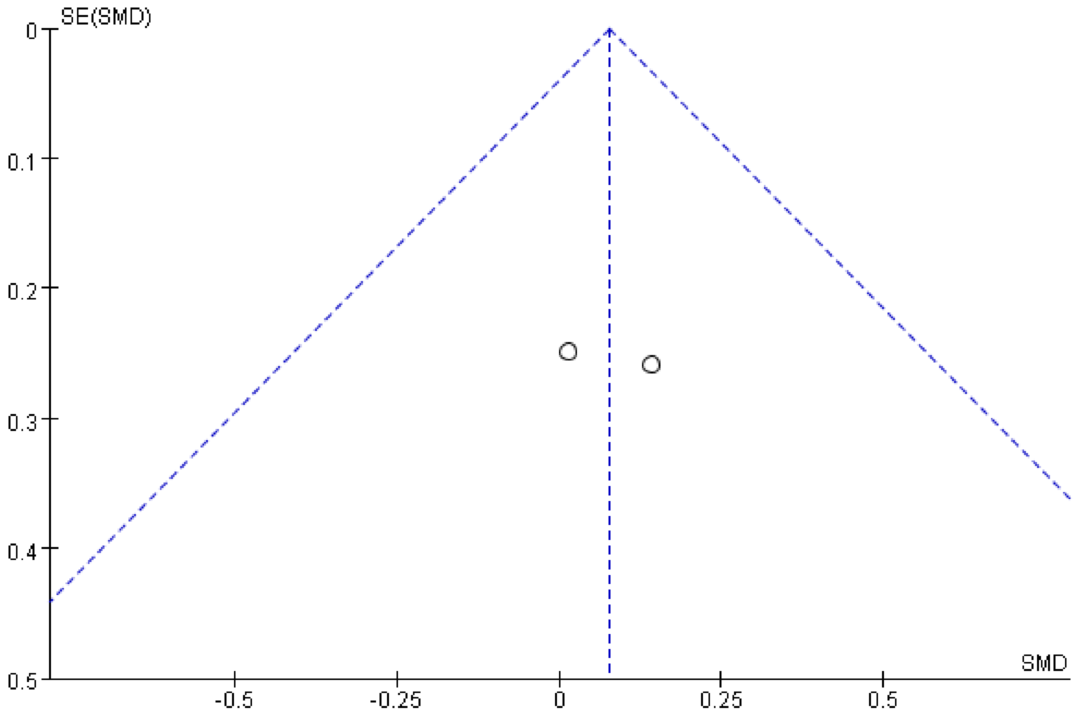

5.2 Postoperative CFSS-DS

Study or Subgroup	Experimental Mean	Experimental SD	Experimental Total	Control Mean	Control SD	Control Total	Weight	Std. Mean Difference IV, Fixed, 95% CI
Jian Yu 2022	28.73	4.92	30	36.9	6.28	30	46.4%	-1.43 [-2.00, -0.86]
Natthatida Janthasila 2022	22.4	7.3	33	33.6	10.8	32	53.6%	-1.20 [-1.74, -0.67]
**Total (95% CI)**			**63**			**62**	**100.0%**	**-1.31 [-1.70, -0.92]**

Heterogeneity: Chi² = 0.32, df = 1 (P = 0.57); I² = 0%
Test for overall effect: Z = 6.60 (P < 0.00001)

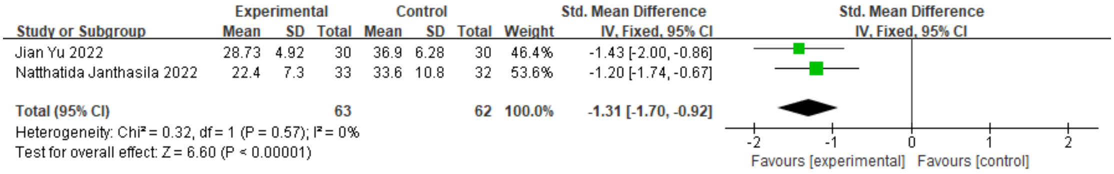

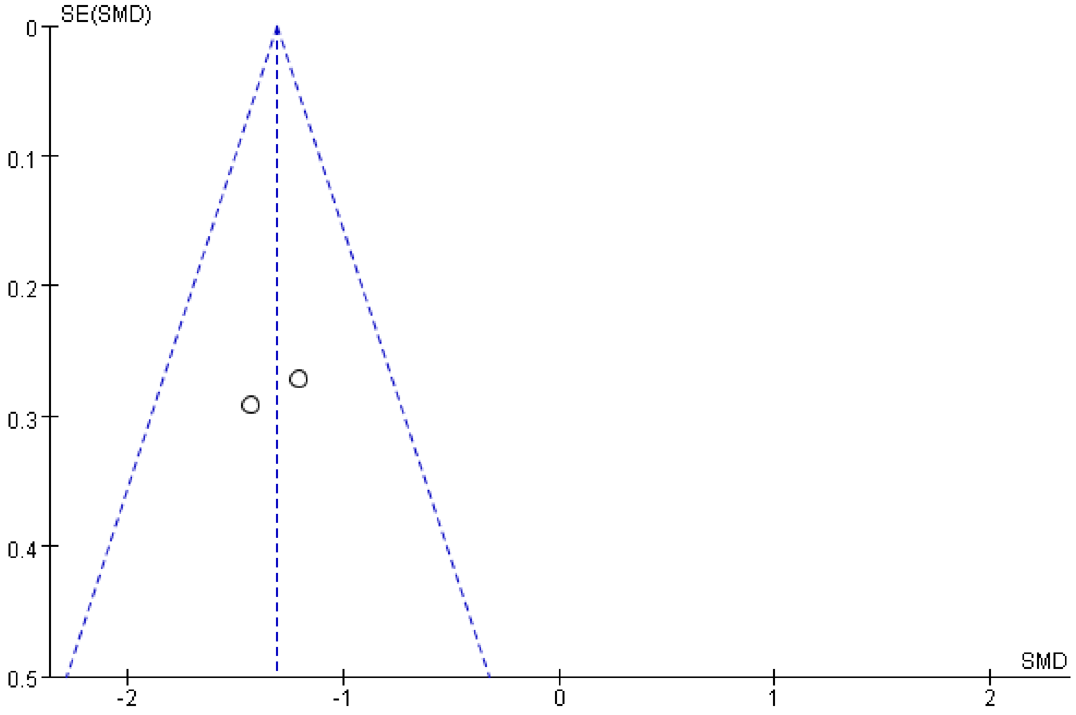

5.3 CFSS-DS Change

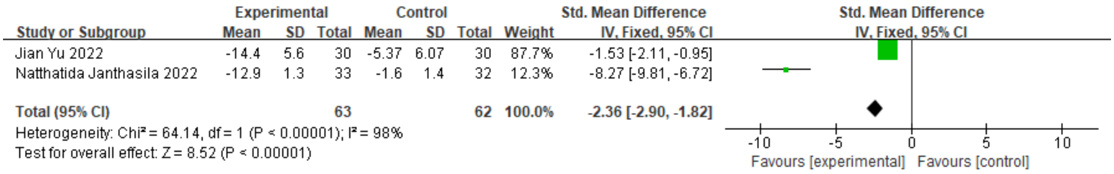

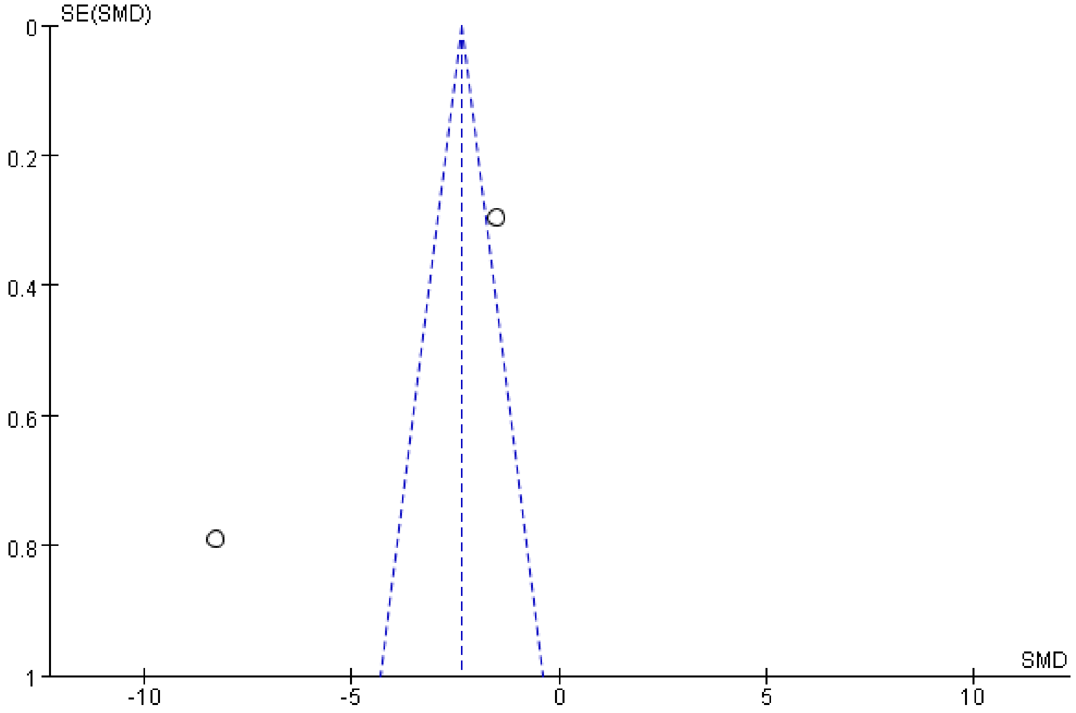

6. MDAS

6.1 Preoperative MDAS

	Experimental			Control				Std. Mean Difference
Study or Subgroup	Mean	SD	Total	Mean	SD	Total	Weight	IV, Fixed, 95% CI
Huijun Hu 2020	14.23	2.35	30	14.34	2.43	28	29.3%	-0.05 [-0.56, 0.47]
Juan Wang 2021	16.23	1.61	30	15.87	1.38	30	30.2%	0.24 [-0.27, 0.74]
NingRuan 2011	14.2	2.34	40	14.37	3.1	40	40.5%	-0.06 [-0.50, 0.38]
**Total (95% CI)**			**100**			**98**	**100.0%**	**0.03 [-0.25, 0.31]**

Heterogeneity: Chi² = 0.89, df = 2 (P = 0.64); I² = 0%
Test for overall effect: Z = 0.23 (P = 0.81)

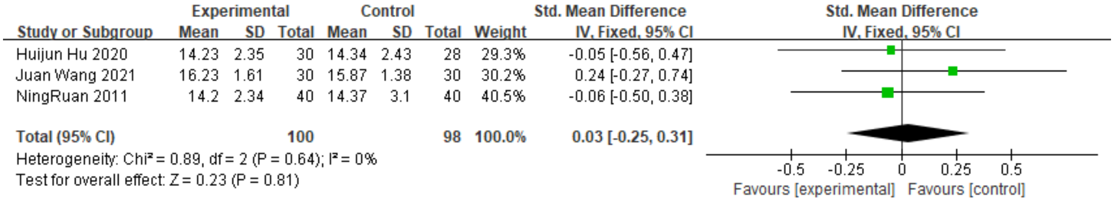

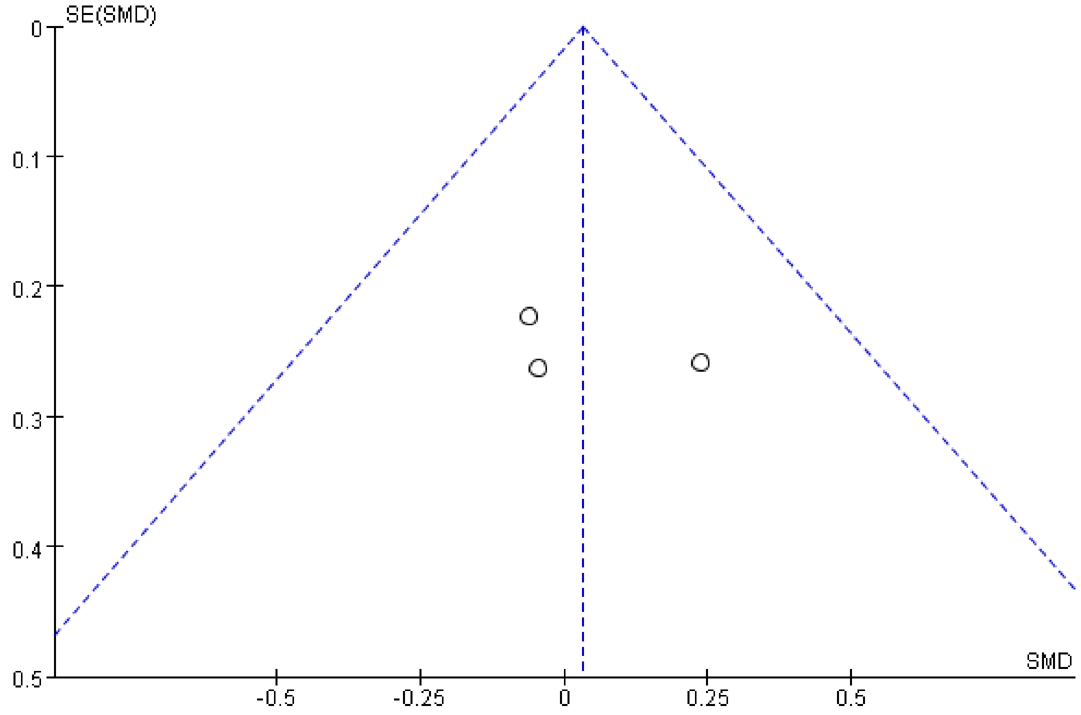

6.2 Postoperative MDAS

	Experimental			Control				Std. Mean Difference
Study or Subgroup	Mean	SD	Total	Mean	SD	Total	Weight	IV, Fixed, 95% CI
Huijun Hu 2020	11.25	1.69	30	14.26	1.74	28	24.4%	-1.73 [-2.34, -1.12]
Juan Wang 2021	12.83	3.43	30	15.8	2.75	30	31.7%	-0.94 [-1.48, -0.41]
NingRuan 2011	11.46	3.02	40	14.24	4.1	40	43.9%	-0.76 [-1.22, -0.31]
**Total (95% CI)**			**100**			**98**	**100.0%**	**-1.06 [-1.36, -0.76]**

Heterogeneity: Chi² = 6.47, df = 2 (P = 0.04); I² = 69%
Test for overall effect: Z = 6.88 (P < 0.00001)

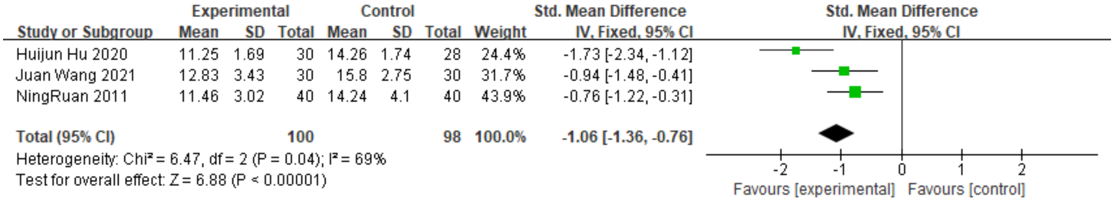

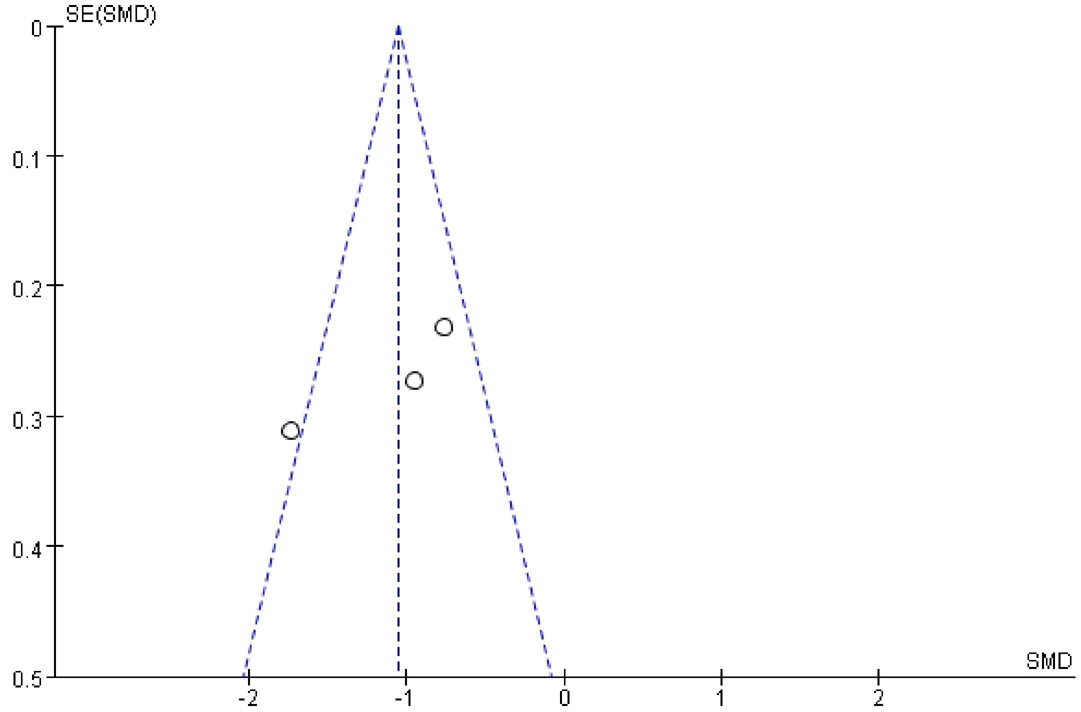

6.3 MDAS Change

Study or Subgroup	Experimental Mean	Experimental SD	Experimental Total	Control Mean	Control SD	Control Total	Weight	Std. Mean Difference IV, Fixed, 95% CI
Huijun Hu 2020	-2.98	2.1	30	-0.08	2.17	28	27.1%	-1.34 [-1.91, -0.77]
Juan Wang 2021	-3.4	3.42	30	-0.07	2.43	30	29.9%	-1.11 [-1.65, -0.56]
NingRuan 2011	-2.74	2.74	40	-0.13	3.7	40	42.9%	-0.79 [-1.25, -0.34]
**Total (95% CI)**			**100**			**98**	**100.0%**	**-1.04 [-1.34, -0.74]**

Heterogeneity: Chi² = 2.23, df = 2 (P = 0.33); I² = 10%
Test for overall effect: Z = 6.80 (P < 0.00001)

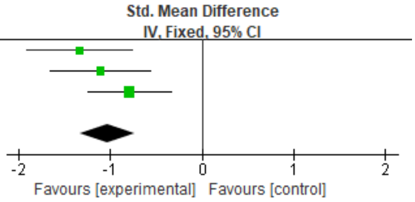

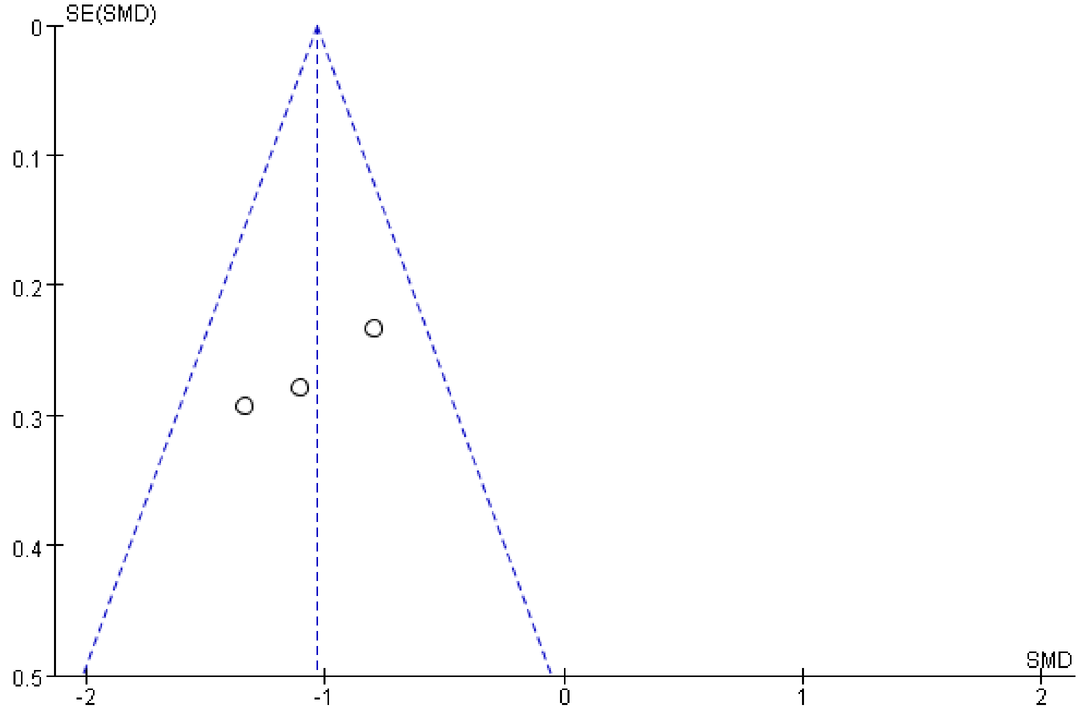

7. DAS

7.1 Preoperative DAS

Study or Subgroup	Experimental Mean	Experimental SD	Experimental Total	Control Mean	Control SD	Control Total	Weight	Std. Mean Difference IV, Fixed, 95% CI
Haiyun Lu 2008	14.1	1.1	50	14.4	1.1	50	31.2%	-0.27 [-0.66, 0.12]
Yu-Kyoung Kim 2011	13.42	3.09	106	13.15	2.87	113	68.8%	0.09 [-0.17, 0.36]
**Total (95% CI)**			**156**			**163**	**100.0%**	**-0.02 [-0.24, 0.20]**

Heterogeneity: Chi² = 2.22, df = 1 (P = 0.14); I² = 55%
Test for overall effect: Z = 0.20 (P = 0.84)

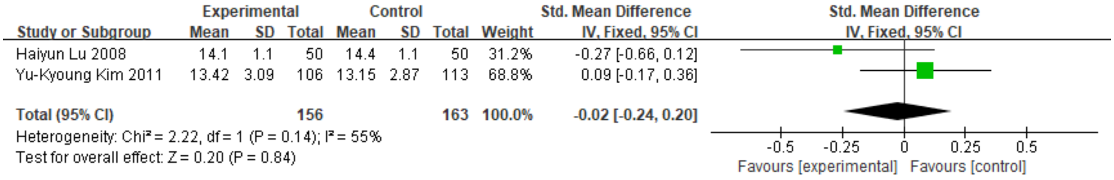

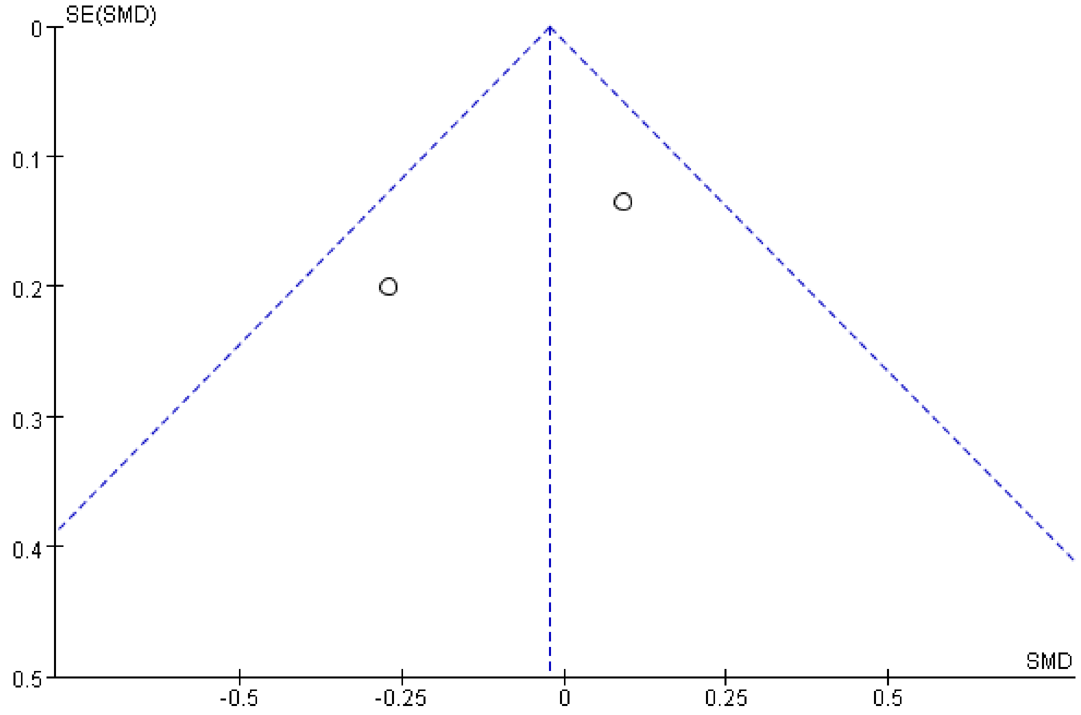

7.2 Postoperative DAS

Study or Subgroup	Experimental Mean	Experimental SD	Experimental Total	Control Mean	Control SD	Control Total	Weight	Std. Mean Difference IV, Fixed, 95% CI
Haiyun Lu 2008	14.29	2	50	14.85	2	50	31.2%	-0.28 [-0.67, 0.12]
Yu-Kyoung Kim 2011	13.12	3.24	106	13.51	3.11	113	68.8%	-0.12 [-0.39, 0.14]
**Total (95% CI)**			**156**			**163**	**100.0%**	**-0.17 [-0.39, 0.05]**

Heterogeneity: Chi² = 0.41, df = 1 (P = 0.52); I² = 0%
Test for overall effect: Z = 1.52 (P = 0.13)

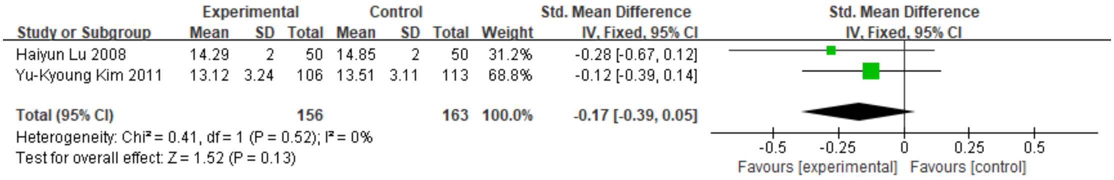

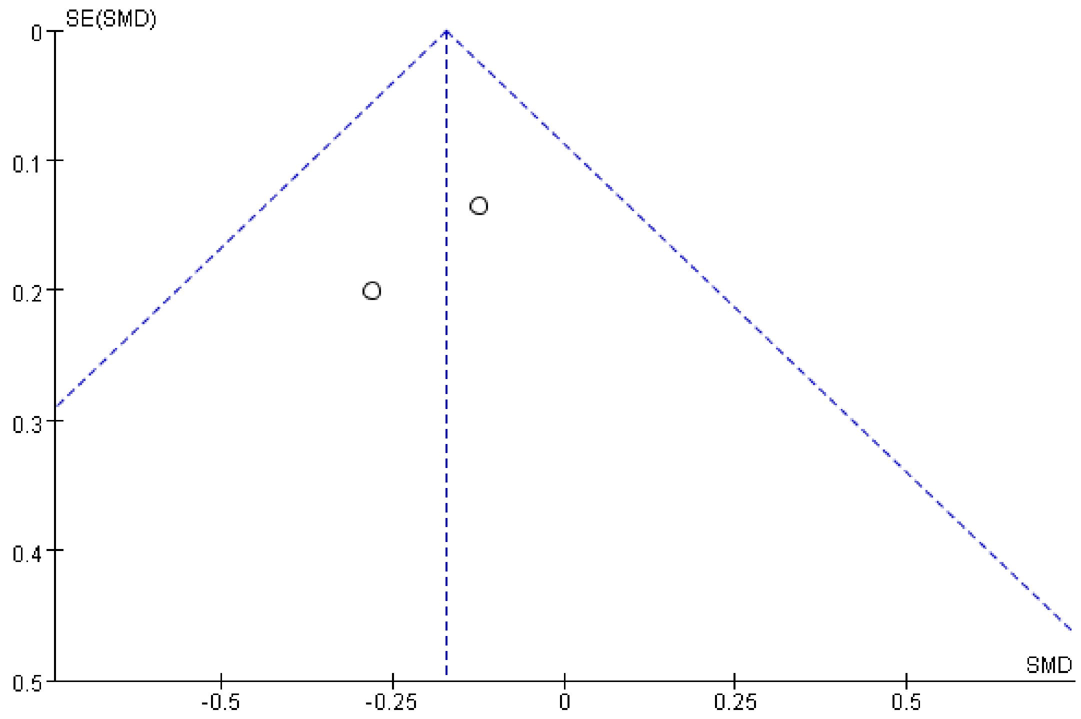

7.3 DAS Change

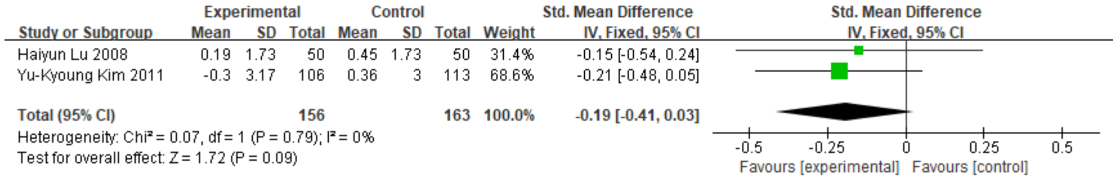

Study or Subgroup	Experimental Mean	Experimental SD	Experimental Total	Control Mean	Control SD	Control Total	Weight	Std. Mean Difference IV, Fixed, 95% CI
Haiyun Lu 2008	0.19	1.73	50	0.45	1.73	50	31.4%	-0.15 [-0.54, 0.24]
Yu-Kyoung Kim 2011	-0.3	3.17	106	0.36	3	113	68.6%	-0.21 [-0.48, 0.05]
**Total (95% CI)**			**156**			**163**	**100.0%**	**-0.19 [-0.41, 0.03]**

Heterogeneity: Chi² = 0.07, df = 1 (P = 0.79); I² = 0%
Test for overall effect: Z = 1.72 (P = 0.09)

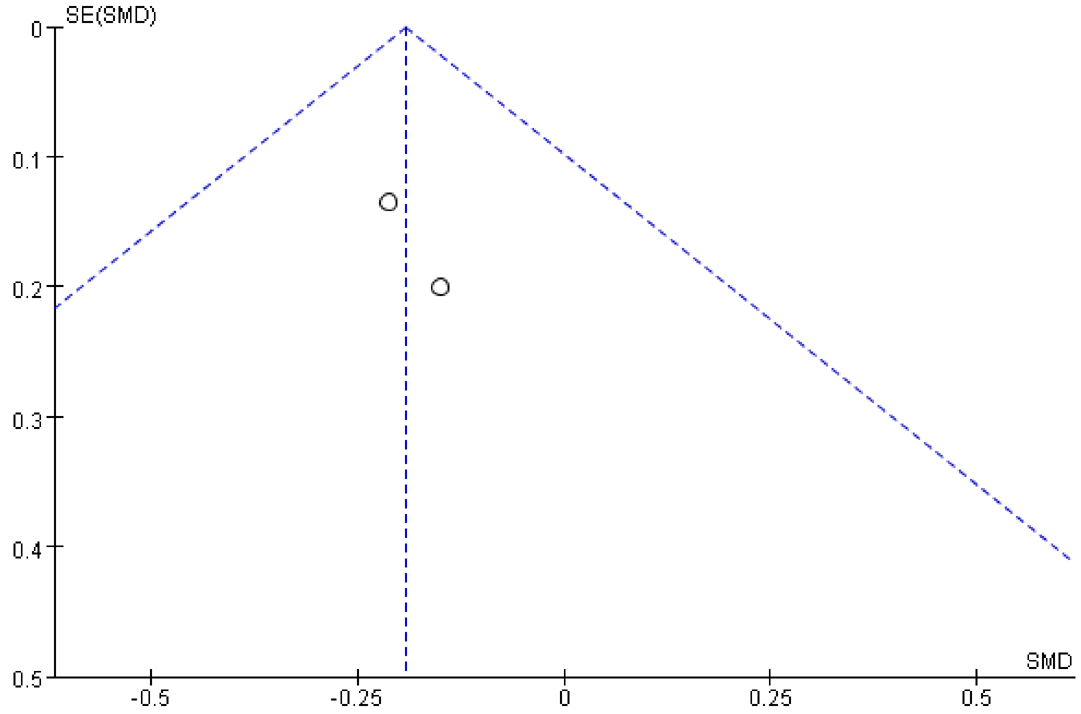

8. S-AI

8.1 Preoperative S-AI

Study or Subgroup	Experimental Mean	Experimental SD	Experimental Total	Control Mean	Control SD	Control Total	Weight	Std. Mean Difference IV, Fixed, 95% CI
Claas Lahmann 2008	41.3	9.6	28	41.9	11.5	30	26.0%	-0.06 [-0.57, 0.46]
Hui-Ling Lai 2008	50.64	5.1	22	49.73	5.49	22	19.7%	0.17 [-0.42, 0.76]
NingRuan 2011	55.18	5.03	40	54.62	4.21	40	35.9%	0.12 [-0.32, 0.56]
Yang Gao 2006	56.38	7.72	21	52.95	8.89	21	18.4%	0.40 [-0.21, 1.02]
**Total (95% CI)**			**111**			**113**	**100.0%**	**0.14 [-0.13, 0.40]**

Heterogeneity: $Chi^2 = 1.29$, df = 3 (P = 0.73); $I^2 = 0\%$
Test for overall effect: Z = 1.02 (P = 0.31)

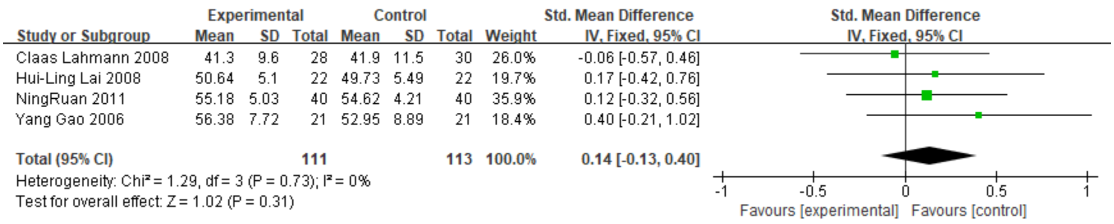

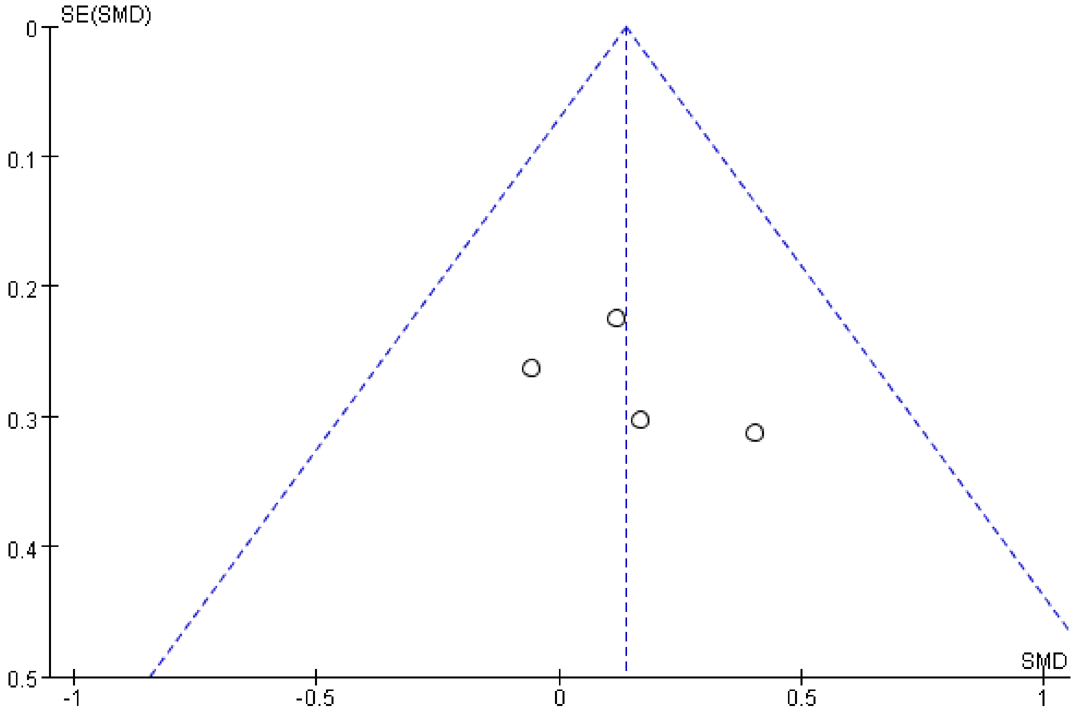

8.2 Postoperative S-AI

Study or Subgroup	Experimental Mean	Experimental SD	Experimental Total	Control Mean	Control SD	Control Total	Weight	Std. Mean Difference IV, Fixed, 95% CI
Claas Lahmann 2008	36.8	9.8	28	40.5	11.2	30	29.7%	-0.35 [-0.87, 0.17]
Hui-Ling Lai 2008	32.8	3.61	22	39.55	5.16	22	17.5%	-1.49 [-2.16, -0.81]
NingRuan 2011	44.77	5.97	40	54.37	9.66	40	35.1%	-1.18 [-1.66, -0.71]
Yang Gao 2006	34.1	6.48	21	43.71	7.83	21	17.7%	-1.31 [-1.98, -0.64]
**Total (95% CI)**			**111**			**113**	**100.0%**	**-1.01 [-1.29, -0.73]**

Heterogeneity: $Chi^2 = 9.50$, df = 3 (P = 0.02); $I^2 = 68\%$
Test for overall effect: Z = 7.01 (P < 0.00001)

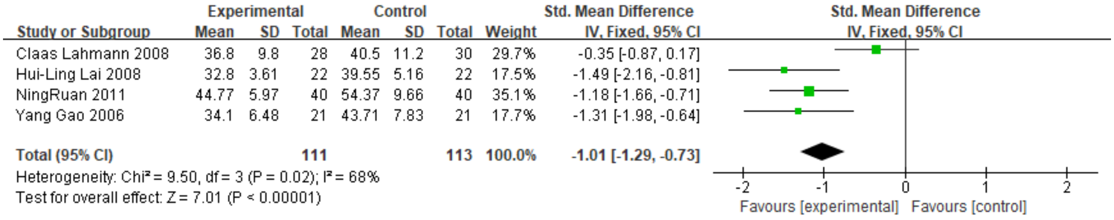

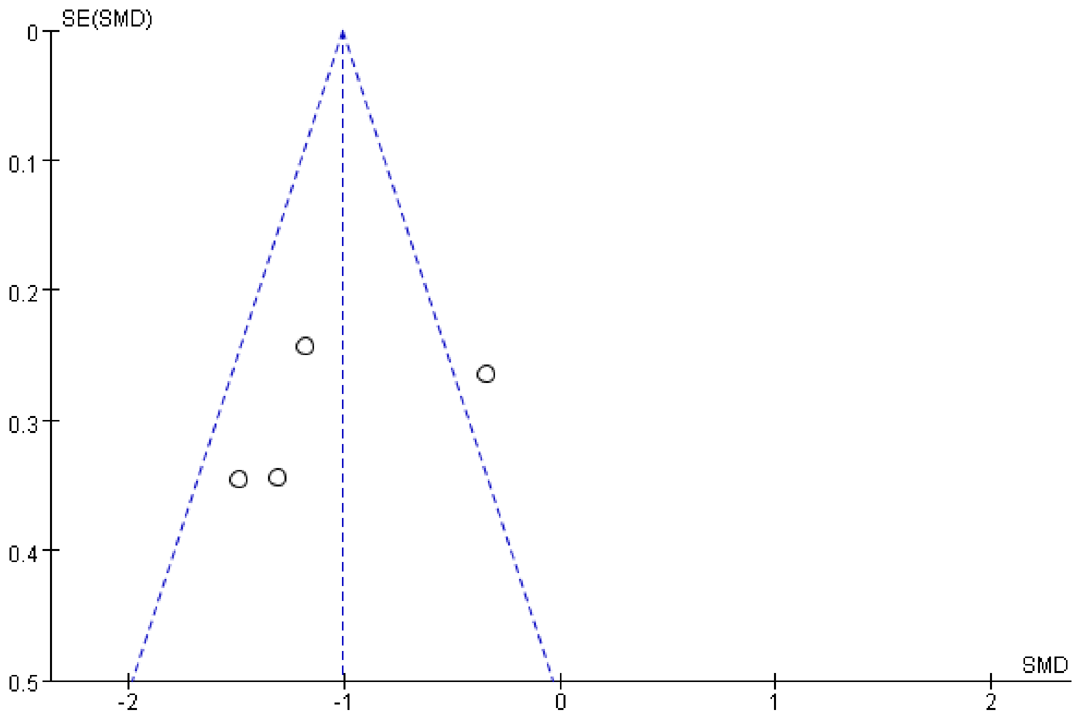

8.3 S-AI Change

Study or Subgroup	Experimental Mean	Experimental SD	Experimental Total	Control Mean	Control SD	Control Total	Weight	Std. Mean Difference IV, Fixed, 95% CI
Claas Lahmann 2008	-4.4	4.6	28	-1.4	4.4	30	32.3%	-0.66 [-1.19, -0.13]
Hui-Ling Lai 2008	-17.84	4.54	22	-10.18	5.33	22	19.7%	-1.52 [-2.20, -0.84]
NingRuan 2011	-10.41	5.56	40	-0.25	8.39	40	37.4%	-1.41 [-1.91, -0.92]
Yang Gao 2006	-22.29	3.7	21	-9.24	4.49	21	10.6%	-3.11 [-4.04, -2.19]
**Total (95% CI)**			**111**			**113**	**100.0%**	**-1.37 [-1.67, -1.07]**

Heterogeneity: Chi² = 20.80, df = 3 (P = 0.0001); I² = 86%
Test for overall effect: Z = 8.92 (P < 0.00001)

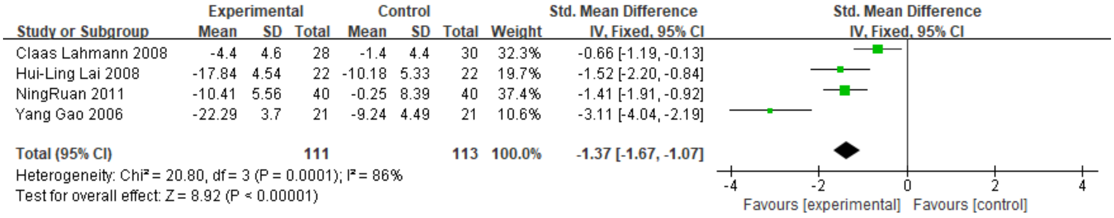

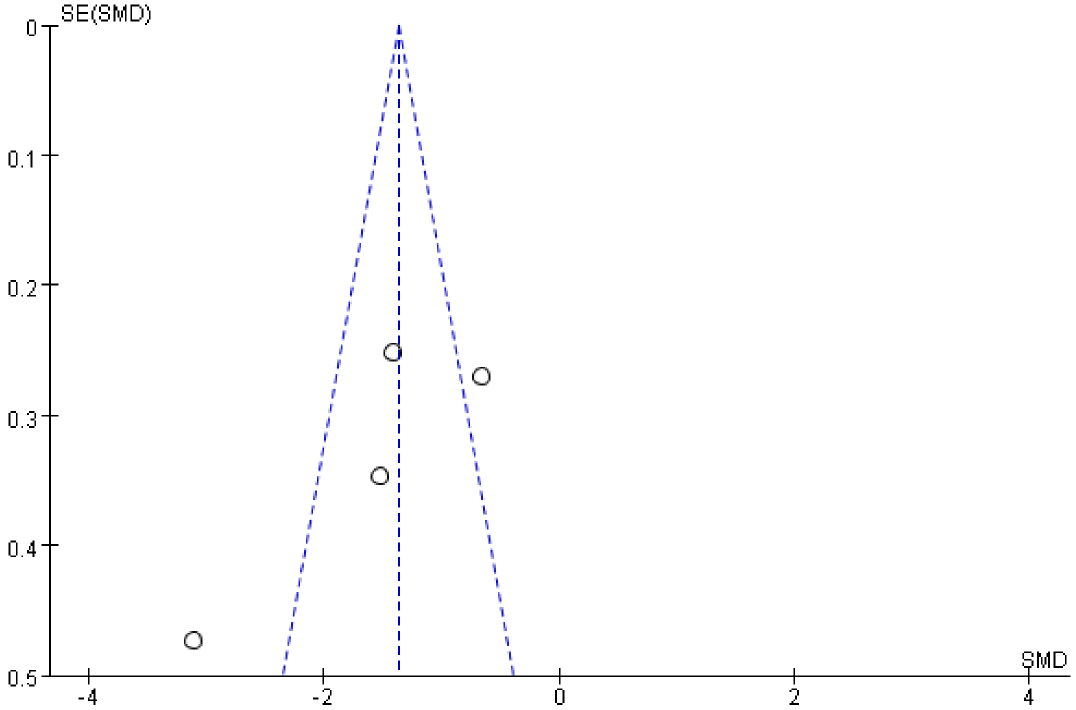

9. VPT

9.1 Preoperative VPT

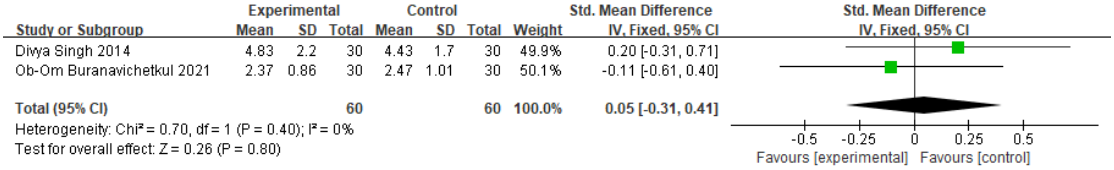

Study or Subgroup	Experimental Mean	Experimental SD	Experimental Total	Control Mean	Control SD	Control Total	Weight	Std. Mean Difference IV, Fixed, 95% CI
Divya Singh 2014	4.83	2.2	30	4.43	1.7	30	49.9%	0.20 [-0.31, 0.71]
Ob-Om Buranavichetkul 2021	2.37	0.86	30	2.47	1.01	30	50.1%	-0.11 [-0.61, 0.40]
**Total (95% CI)**			**60**			**60**	**100.0%**	**0.05 [-0.31, 0.41]**

Heterogeneity: Chi² = 0.70, df = 1 (P = 0.40); I² = 0%
Test for overall effect: Z = 0.26 (P = 0.80)

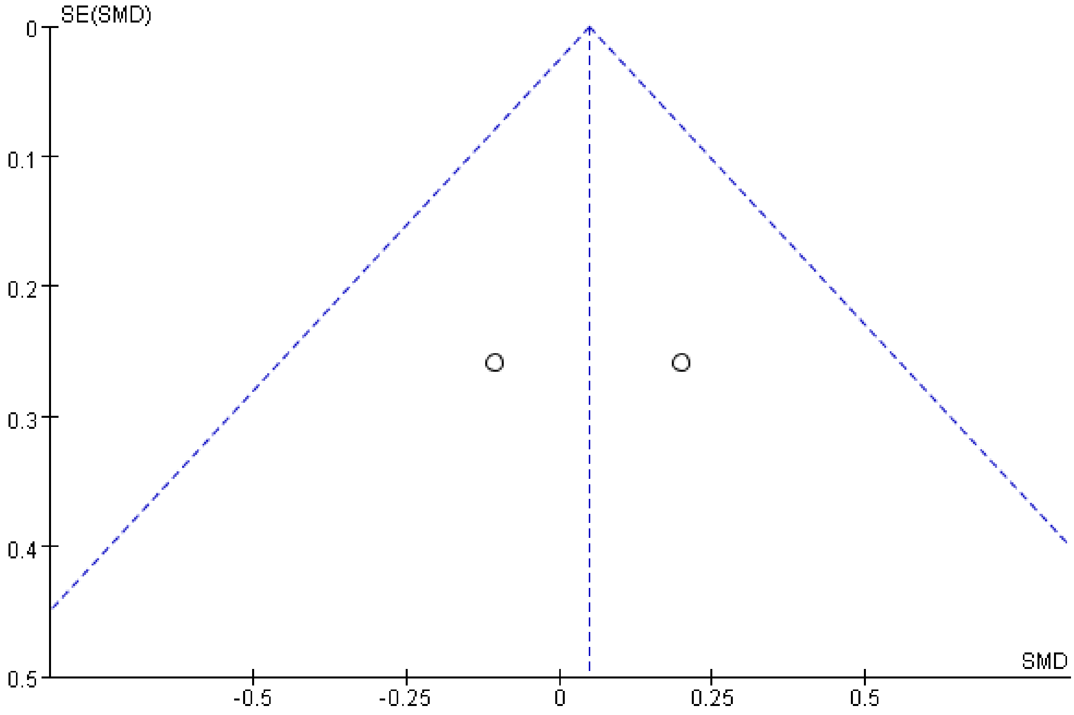

9.2 Postoperative VPT

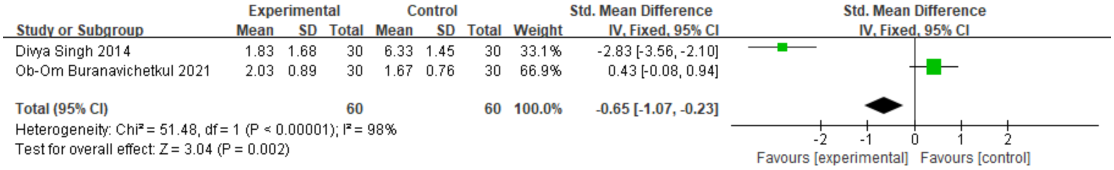

Study or Subgroup	Experimental Mean	Experimental SD	Experimental Total	Control Mean	Control SD	Control Total	Weight	Std. Mean Difference IV, Fixed, 95% CI
Divya Singh 2014	1.83	1.68	30	6.33	1.45	30	33.1%	-2.83 [-3.56, -2.10]
Ob-Om Buranavichetkul 2021	2.03	0.89	30	1.67	0.76	30	66.9%	0.43 [-0.08, 0.94]
**Total (95% CI)**			**60**			**60**	**100.0%**	**-0.65 [-1.07, -0.23]**

Heterogeneity: Chi² = 51.48, df = 1 (P < 0.00001); I² = 98%
Test for overall effect: Z = 3.04 (P = 0.002)

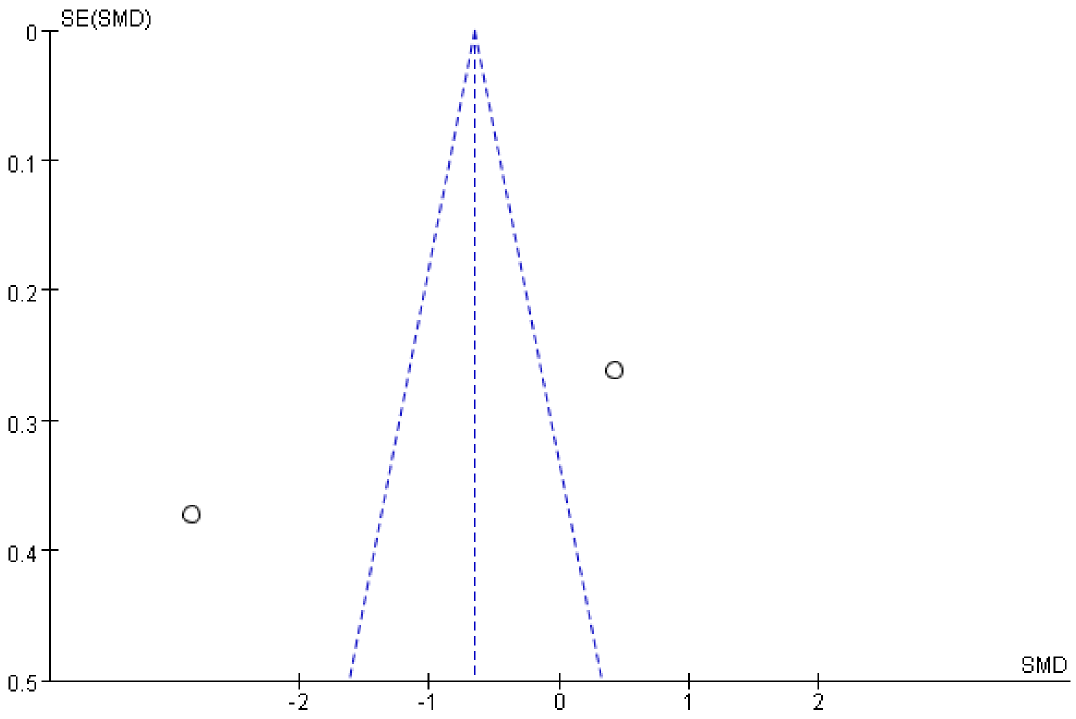

9.3 VPT Change

Study or Subgroup	Experimental Mean	Experimental SD	Experimental Total	Control Mean	Control SD	Control Total	Weight	Std. Mean Difference IV, Fixed, 95% CI
Divya Singh 2014	-3	2.15	30	1.9	1.32	30	34.3%	-2.71 [-3.42, -2.00]
Ob-Om Buranavichetkul 2021	-0.34	0.88	30	-0.8	0.91	30	65.7%	0.51 [-0.01, 1.02]
**Total (95% CI)**			**60**			**60**	**100.0%**	**-0.60 [-1.01, -0.18]**

Heterogeneity: Chi² = 51.48, df = 1 (P < 0.00001); I² = 98%
Test for overall effect: Z = 2.80 (P = 0.005)

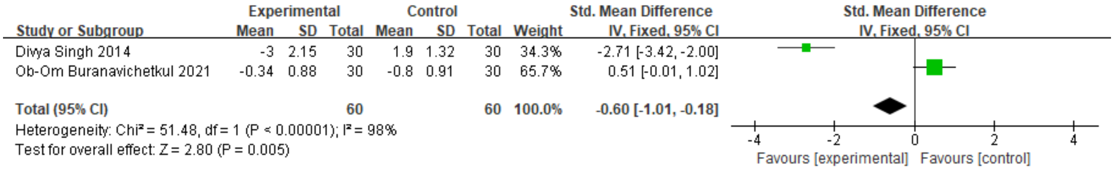

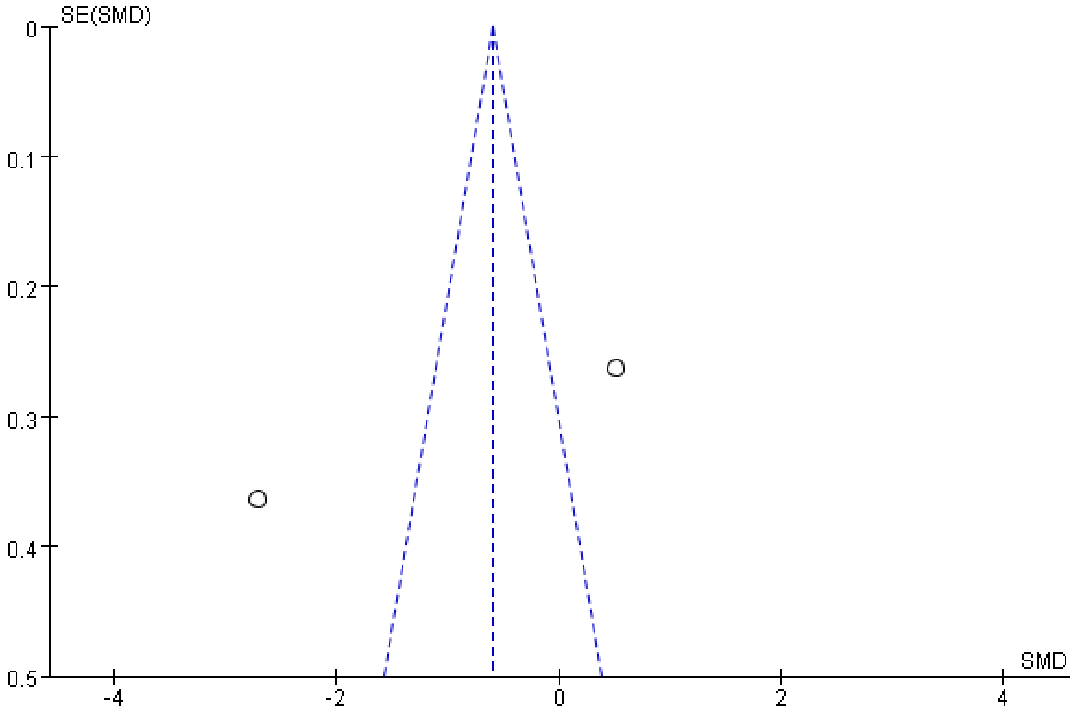

10. Pain Score

10.1 Preoperative Pain Score

Study or Subgroup	Experimental Mean	Experimental SD	Experimental Total	Control Mean	Control SD	Control Total	Weight	Std. Mean Difference IV, Fixed, 95% CI
Huijun Hu 2020	6.12	2.18	30	6.18	2.14	28	20.8%	-0.03 [-0.54, 0.49]
Juan Wang 2021	3.02	2.2	30	2.91	1.28	30	21.5%	0.06 [-0.45, 0.57]
NingRuan 2011	7.2	1.83	40	6.63	2.18	40	28.4%	0.28 [-0.16, 0.72]
Serge Kalongo TSHISWAKA 2020	1.6	1.66	20	2.1	3.4	20	14.3%	-0.18 [-0.80, 0.44]
Yang Gao 2006	7.98	1.67	21	7.71	1.67	21	15.0%	0.16 [-0.45, 0.76]
**Total (95% CI)**			**141**			**139**	**100.0%**	**0.08 [-0.15, 0.32]**

Heterogeneity: Chi² = 1.72, df = 4 (P = 0.79); I² = 0%
Test for overall effect: Z = 0.71 (P = 0.48)

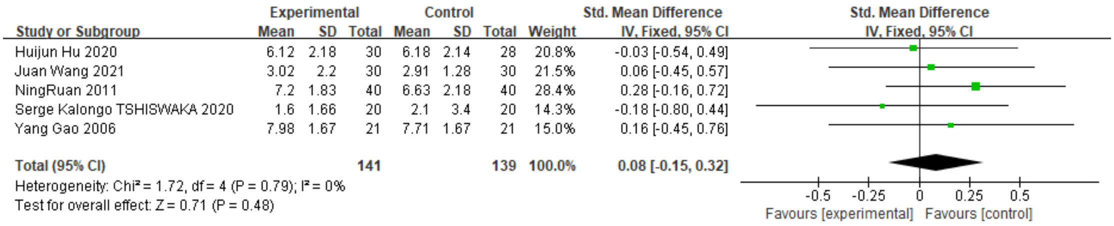

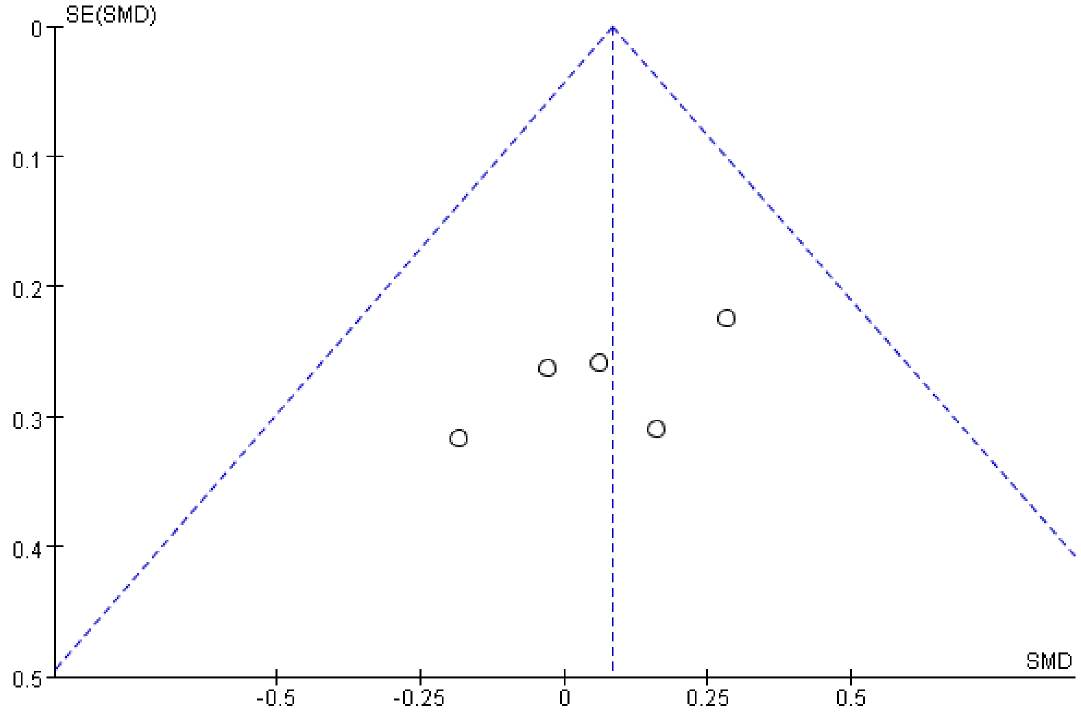

10.2 Postoperative Pain Score

Study or Subgroup	Experimental Mean	Experimental SD	Experimental Total	Control Mean	Control SD	Control Total	Weight	Std. Mean Difference IV, Fixed, 95% CI
Huijun Hu 2020	2.64	1.54	30	5.98	1.67	28	15.3%	-2.05 [-2.70, -1.41]
Juan Wang 2021	1.23	1.3	30	3.12	1.44	30	19.9%	-1.36 [-1.93, -0.79]
NingRuan 2011	5.4	2.51	40	6.27	2	40	32.5%	-0.38 [-0.82, 0.06]
Serge Kalongo TSHISWAKA 2020	1	1.52	20	1.1	2.38	20	16.6%	-0.05 [-0.67, 0.57]
Yang Gao 2006	2.55	1.32	21	3.88	1.56	21	15.6%	-0.90 [-1.54, -0.27]
**Total (95% CI)**			**141**			**139**	**100.0%**	**-0.86 [-1.11, -0.61]**

Heterogeneity: Chi² = 27.32, df = 4 (P < 0.0001); I² = 85%
Test for overall effect: Z = 6.67 (P < 0.00001)

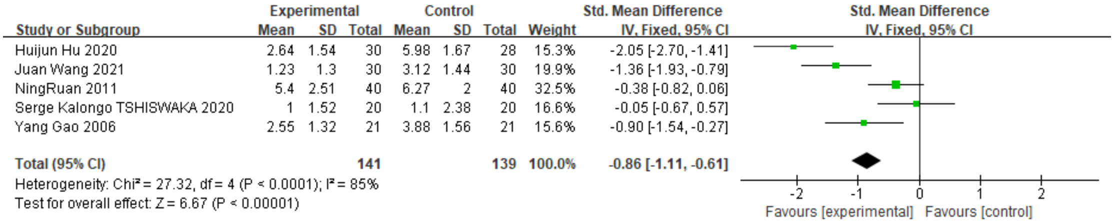

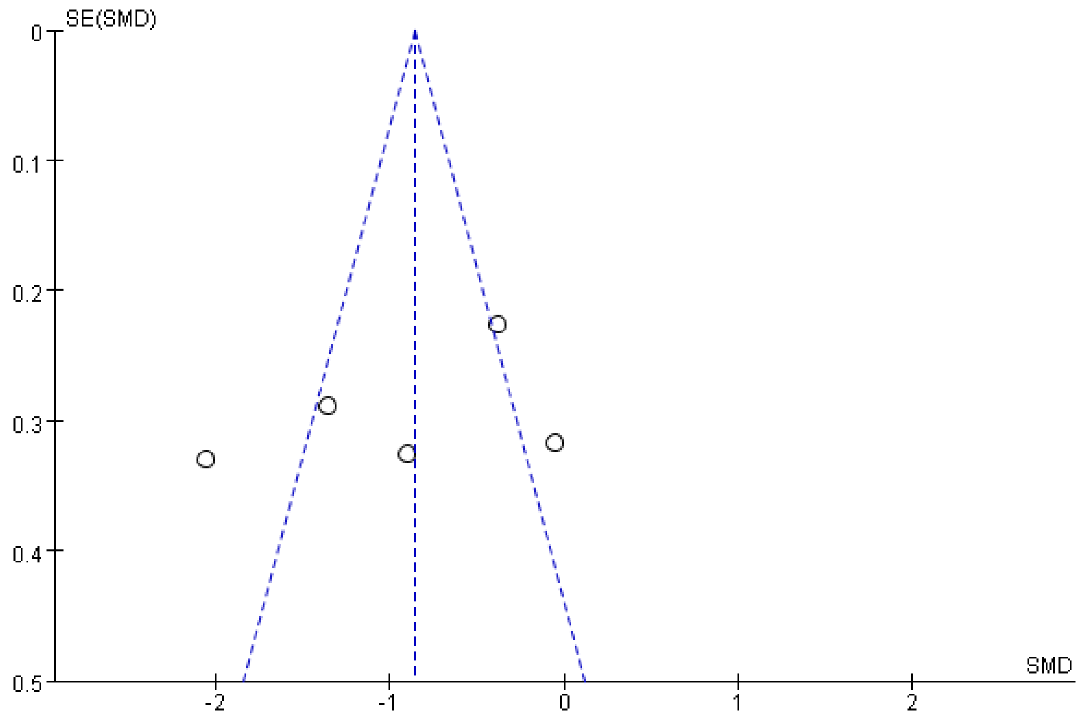

10.3 Pain Score Change

Study or Subgroup	Experimental Mean	Experimental SD	Experimental Total	Control Mean	Control SD	Control Total	Weight	Std. Mean Difference IV, Fixed, 95% CI
Huijun Hu 2020	-3.48	1.94	30	-0.2	1.95	28	17.1%	-1.66 [-2.27, -1.06]
Juan Wang 2021	-1.78	2.39	30	0.21	1.02	30	21.0%	-1.07 [-1.61, -0.53]
NingRuan 2011	-1.8	2.25	40	-0.36	2.1	40	30.6%	-0.66 [-1.11, -0.20]
Serge Kalongo TSHISWAKA 2020	-0.6	1.59	20	-1	3.02	20	16.1%	0.16 [-0.46, 0.78]
Yang Gao 2006	-5.43	1.75	21	-3.88	1.56	21	15.2%	-0.92 [-1.56, -0.28]
**Total (95% CI)**			**141**			**139**	**100.0%**	**-0.82 [-1.07, -0.57]**

Heterogeneity: Chi² = 18.55, df = 4 (P = 0.0010); I² = 78%
Test for overall effect: Z = 6.47 (P < 0.00001)

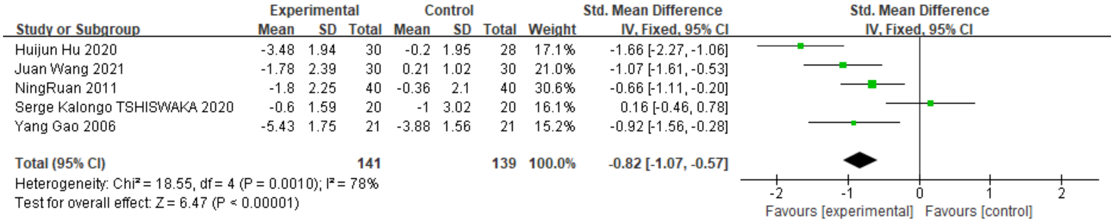

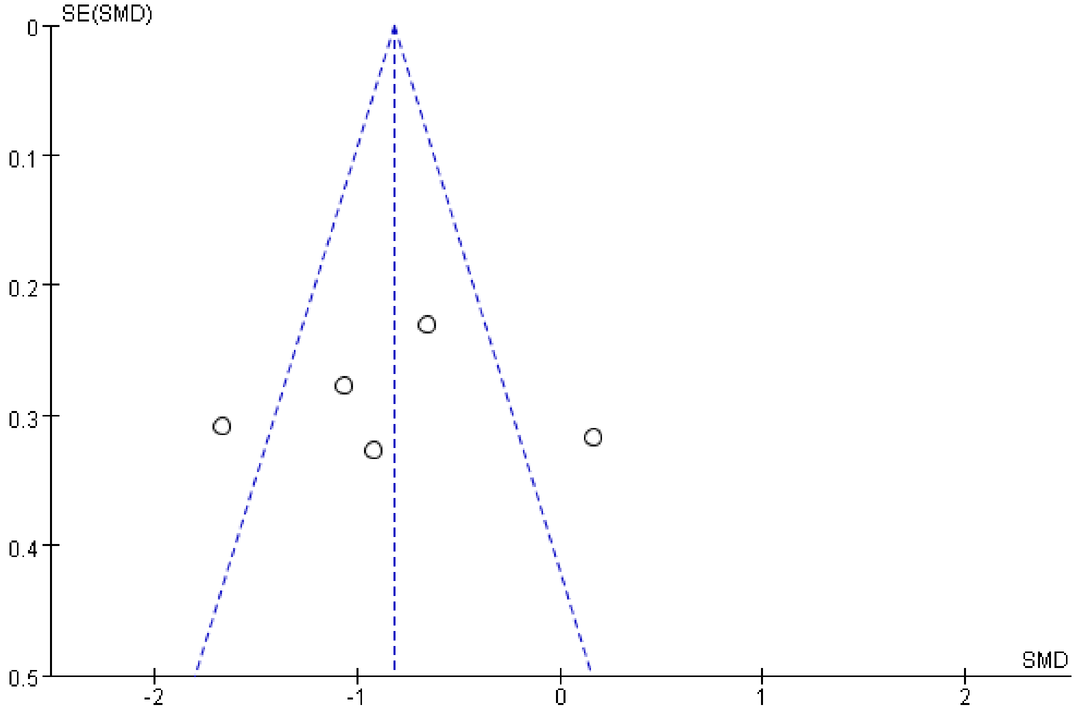

Subject-based subgroup analysis (adults and children)

1. Heart Rate

Study or Subgroup	Experimental Mean	Experimental SD	Experimental Total	Control Mean	Control SD	Control Total	Weight	Std. Mean Difference IV, Fixed, 95% CI
**1.1.1 Child**								
Divya Singh 2014	-13.7	12.8	30	5	17.4	30	5.4%	-1.21 [-1.76, -0.66]
Jeswin James 2021	-9.48	11.97	50	4.8	12.04	50	9.2%	-1.18 [-1.61, -0.75]
Natthatida Janthasila 2022	-1	1.5	33	6.5	1.4	32	1.6%	-5.10 [-6.13, -4.08]
Serge Kalongo TSHISWAKA 2020	-8.8	15.31	20	-4.2	14.95	20	4.3%	-0.30 [-0.92, 0.33]
Uma B Dixit 2020	-2.6	13.55	40	0.3	12.22	40	8.6%	-0.22 [-0.66, 0.22]
Yizhu Wang 2018	1.44	15.92	64	18.38	40.62	64	13.4%	-0.55 [-0.90, -0.19]
**Subtotal (95% CI)**			**237**			**236**	**42.5%**	**-0.85 [-1.04, -0.65]**
Heterogeneity: Chi² = 83.45, df = 5 (P < 0.00001); I² = 94%								
Test for overall effect: Z = 8.38 (P < 0.00001)								
**1.1.2 Adult**								
Cynthia Mejía-Rubalcava 2015	-7.7	9.1	17	3.4	6.36	17	2.9%	-1.38 [-2.14, -0.62]
Haiyun Lu 2008	1.4	5.11	50	7.5	4.8	50	9.1%	-1.22 [-1.65, -0.79]
Hui-Ling Lai 2008	-5.68	6.21	22	-4.59	7.41	22	4.8%	-0.16 [-0.75, 0.44]
Ob-Om Buranavichetkul 2021	-8.4	12.31	30	-9.04	14.71	30	6.5%	0.05 [-0.46, 0.55]
Xinzhi Yue 2013	2.1	6.56	49	4.1	6.24	49	10.5%	-0.31 [-0.71, 0.09]
Yu-Kyoung Kim 2011	-15.76	8.16	106	-14.65	12.43	113	23.7%	-0.10 [-0.37, 0.16]
**Subtotal (95% CI)**			**274**			**281**	**57.5%**	**-0.37 [-0.54, -0.20]**
Heterogeneity: Chi² = 29.04, df = 5 (P < 0.0001); I² = 83%								
Test for overall effect: Z = 4.26 (P < 0.0001)								
**Total (95% CI)**			**511**			**517**	**100.0%**	**-0.57 [-0.70, -0.44]**
Heterogeneity: Chi² = 125.26, df = 11 (P < 0.00001); I² = 91%								
Test for overall effect: Z = 8.69 (P < 0.00001)								
Test for subgroup differences: Chi² = 12.77, df = 1 (P = 0.0004), I² = 92.2%								

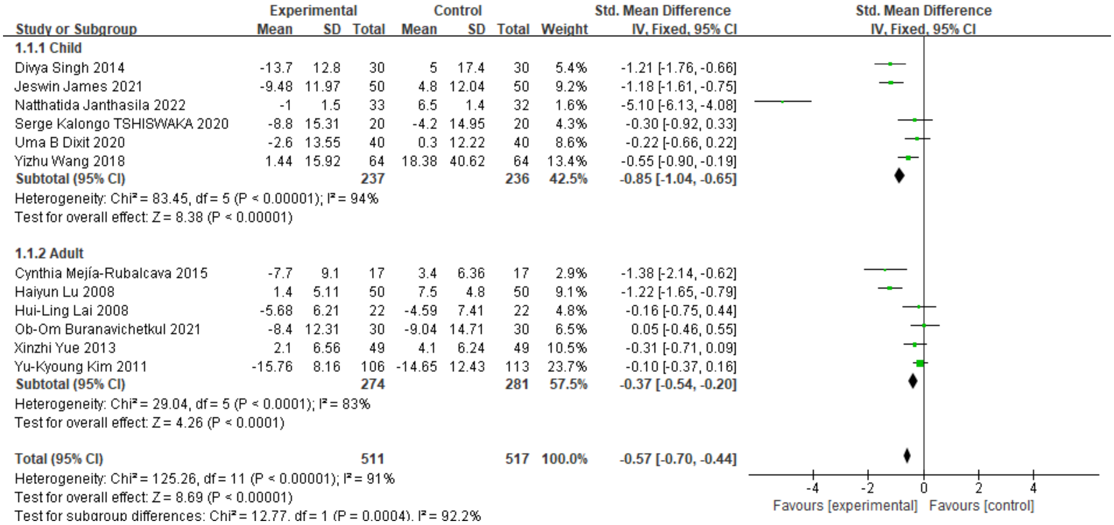

2. Systolic Blood Pressure

Study or Subgroup	Experimental Mean	Experimental SD	Experimental Total	Control Mean	Control SD	Control Total	Weight	Std. Mean Difference IV, Fixed, 95% CI
**2.17.1 Child**								
Divya Singh 2014	118.8	12.1	30	127.8	9.5	30	6.6%	-0.82 [-1.34, -0.29]
Natthatida Janthasila 2022	101.2	11.3	33	106.5	11.7	32	7.5%	-0.46 [-0.95, 0.04]
Uma B Dixit 2020	110	7.1	40	112.2	8.5	40	9.4%	-0.28 [-0.72, 0.16]
Yizhu Wang 2018	99.89	6.58	64	110.94	11.99	64	13.1%	-1.14 [-1.51, -0.76]
**Subtotal (95% CI)**			**167**			**166**	**36.6%**	**-0.72 [-0.94, -0.49]**
Heterogeneity: Chi² = 9.84, df = 3 (P = 0.02); I² = 70%								
Test for overall effect: Z = 6.29 (P < 0.00001)								
**2.17.2 Adult**								
Cynthia Mejía-Rubalcava 2015	122.5	7.5	17	134.9	6.2	17	2.8%	-1.76 [-2.57, -0.95]
Haiyun Lu 2008	108.1	7.1	50	110.6	4	50	11.6%	-0.43 [-0.83, -0.03]
Hui-Ling Lai 2008	127.5	11.1	22	145.45	14.76	22	4.2%	-1.35 [-2.01, -0.69]
Ob-Om Buranavichetkul 2021	122.3	26.65	30	127.3	16.56	30	7.1%	-0.22 [-0.73, 0.29]
Xinzhi Yue 2013	122	5	49	123.7	5.4	49	11.5%	-0.32 [-0.72, 0.07]
Yu-Kyoung Kim 2011	118.61	12.36	106	117.92	10.45	113	26.1%	0.06 [-0.20, 0.33]
**Subtotal (95% CI)**			**274**			**281**	**63.4%**	**-0.31 [-0.48, -0.14]**
Heterogeneity: Chi² = 29.90, df = 5 (P < 0.0001); I² = 83%								
Test for overall effect: Z = 3.53 (P = 0.0004)								
**Total (95% CI)**			**441**			**447**	**100.0%**	**-0.46 [-0.59, -0.32]**
Heterogeneity: Chi² = 47.99, df = 9 (P < 0.00001); I² = 81%								
Test for overall effect: Z = 6.61 (P < 0.00001)								
Test for subgroup differences: Chi² = 8.25, df = 1 (P = 0.004), I² = 87.9%								

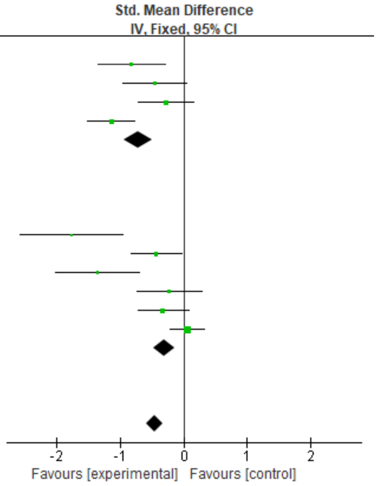

3. Diastolic Blood Pressure

Study or Subgroup	Experimental Mean	Experimental SD	Experimental Total	Control Mean	Control SD	Control Total	Weight	Std. Mean Difference IV, Fixed, 95% CI
**2.18.1 Child**								
Divya Singh 2014	78.2	12.5	30	83.4	8	30	7.6%	-0.49 [-1.00, 0.03]
Natthatida Janthasila 2022	67.3	7.9	33	69.2	9.8	32	8.5%	-0.21 [-0.70, 0.28]
Yizhu Wang 2018	60.55	8.19	64	70.39	15.14	64	15.5%	-0.80 [-1.16, -0.44]
**Subtotal (95% CI)**			**127**			**126**	**31.7%**	**-0.57 [-0.82, -0.32]**
Heterogeneity: Chi² = 3.79, df = 2 (P = 0.15); I² = 47%								
Test for overall effect: Z = 4.41 (P < 0.0001)								
**2.18.2 Adult**								
Cynthia Mejía-Rubalcava 2015	74.9	7.5	17	88.8	6.5	17	2.9%	-1.93 [-2.77, -1.10]
Haiyun Lu 2008	67.4	3	50	70.3	3.1	50	11.8%	-0.94 [-1.36, -0.53]
Hui-Ling Lai 2008	84.91	9.54	22	80.5	7.71	22	5.6%	0.50 [-0.10, 1.10]
Ob-Om Buranavichetkul 2021	73.57	9.94	30	76.5	9.25	30	7.8%	-0.30 [-0.81, 0.21]
Xinzhi Yue 2013	73	5	49	77.3	5.2	49	11.8%	-0.84 [-1.25, -0.42]
Yu-Kyoung Kim 2011	74.63	5.46	106	77.38	11.2	113	28.4%	-0.31 [-0.57, -0.04]
**Subtotal (95% CI)**			**274**			**281**	**68.3%**	**-0.51 [-0.68, -0.34]**
Heterogeneity: Chi² = 31.56, df = 5 (P < 0.00001); I² = 84%								
Test for overall effect: Z = 5.83 (P < 0.00001)								
**Total (95% CI)**			**401**			**407**	**100.0%**	**-0.53 [-0.67, -0.39]**
Heterogeneity: Chi² = 35.48, df = 8 (P < 0.0001); I² = 77%								
Test for overall effect: Z = 7.30 (P < 0.00001)								
Test for subgroup differences: Chi² = 0.13, df = 1 (P = 0.71), I² = 0%								

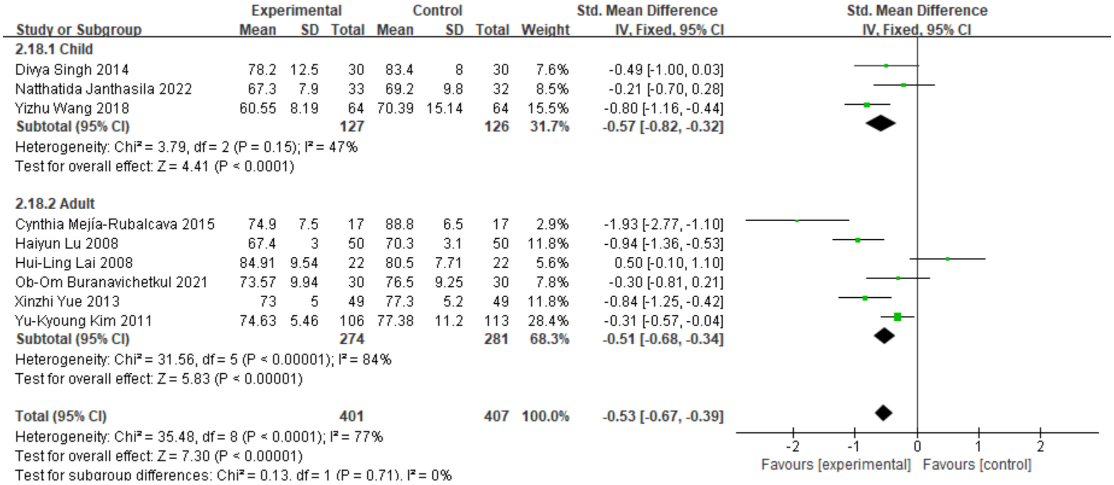

Supplement: Supplementary file 1 [file medicina-59-00209-s001.zip › Supplementary file S3.pdf]
