# Supplementary material for: Efficacy of Music Intervention for Dental Anxiety Disorders: A Systematic Review and Meta-Analysis
Source: Medicina (Kaunas). 2023 Jan 20;59(2):209. doi: 10.3390/medicina59020209 (PMC9961880; doi:10.3390/medicina59020209)
Supplement: Supplementary file 1 [file medicina-59-00209-s001.zip › Supplementary file S4.pdf]

Postoperative Heart Rate

```
tab year, gen(dummy_year)
```

Year	Freq.	Percent	Cum.
2008	2	16.67	16.67
2011	1	8.33	25.00
2013	1	8.33	33.33
2014	1	8.33	41.67
2015	1	8.33	50.00
2018	1	8.33	58.33
2020	2	16.67	75.00
2021	2	16.67	91.67
2022	1	8.33	100.00
Total	12	100.00	

```
. metareg _ES dummy_year1 dummy_year2 dummy_year3 dummy_year4 dummy_year5 dummy_year6 dummy_year7 dummy_year8 dummy_year9, wsse(_ES) b
> sest(reml)
Iteration 1: tau^2 = 0

Meta-analysis regression                          No of studies =    12
                                                  tau^2 method       reml
                                                  tau^2 estimate =      0

Successive values of tau^2 differ by less than 10^-4 :convergence achieved
```

	Coefficient	Std. err.	z	P>|z|	[95% conf.	interval]
dummy_year1	11.37501	17.23279	0.66	0.509	-22.40063	45.15066
dummy_year2	13.3	17.16071	0.78	0.438	-20.33438	46.93439
dummy_year3	13.7	17.08362	0.80	0.423	-19.78329	47.18328
dummy_year4	-3.5	26.35015	-0.13	0.894	-55.14534	48.14534
dummy_year5	(dropped)					
dummy_year6	12.41	17.36411	0.71	0.475	-21.62302	46.44303
dummy_year7	14.2053	16.91834	0.84	0.401	-18.95403	47.36463
dummy_year8	16.46131	16.80324	0.98	0.327	-16.47244	49.39506
dummy_year9	7.900002	19.01184	0.42	0.678	-29.36252	45.16253
_cons	-16.8	16.8	-1.00	0.317	-49.72741	16.1274

```
. tab country, gen(dummy_country)
```

Country	Freq.	Percent	Cum.
Brazil	1	8.33	8.33
China	4	33.33	41.67
India	3	25.00	66.67
Korea	1	8.33	75.00
Mexico	1	8.33	83.33
Thailand	2	16.67	100.00
Total	12	100.00	

```
. metareg _ES dummy_country1 dummy_country2 dummy_country3 dummy_country4 dummy_country5 dummy_country6, wsse(_ES) bsest(reml)
Iteration 1: tau^2 = 0

Meta-analysis regression                          No of studies =    12
                                                  tau^2 method       reml
                                                  tau^2 estimate =      0

Successive values of tau^2 differ by less than 10^-4 :convergence achieved
```

	Coefficient	Std. err.	z	P>|z|	[95% conf.	interval]
dummy_coun~1	5.133512	5.256586	0.98	0.329	-5.169207	15.43623
dummy_coun~2	3.179273	5.241818	0.61	0.544	-7.094502	13.45305
dummy_coun~3	(dropped)					
dummy_coun~4	3.783514	5.937945	0.64	0.524	-7.854645	15.42167
dummy_coun~5	-9.516489	17.47139	-0.54	0.586	-43.75978	24.7268
dummy_coun~6	6.941746	4.808112	1.44	0.149	-2.481981	16.36547
_cons	-7.283514	4.79679	-1.52	0.129	-16.68505	2.118021

```
. tab adult, gen(dummy_adult)
```

Adult	Freq.	Percent	Cum.
Adult	5	41.67	41.67
Children	6	50.00	91.67
Unknown	1	8.33	100.00
Total	12	100.00	

```
. metareg _ES dummy_adult1 dummy_adult2 dummy_adult3, wsse(_ES) bsest(reml)
Iteration 1: tau^2 = 0

Meta-analysis regression                              No of studies =   12
                                                      tau^2 method      reml
                                                      tau^2 estimate =     0

Successive values of tau^2 differ by less than 10^-4 :convergence achieved
```

	Coefficient	Std. err.	z	P>|z|	[95% conf.	interval]
dummy_adult1	5.186743	5.609481	0.92	0.355	-5.807638	16.18112
dummy_adult2	2.140886	5.868857	0.36	0.715	-9.361862	13.64363
dummy_adult3	(dropped)					
_cons	-5.599998	5.599998	-1.00	0.317	-16.57579	5.375797

```
. tab operation, gen(dummy_operation)
```

Operation	Freq.	Percent	Cum.
Oral Therapy	3	25.00	25.00
Pediatric Oral Therapy	5	41.67	66.67
Periodontal surgery	1	8.33	75.00
Root canal therapy	1	8.33	83.33
Tooth extraction	2	16.67	100.00
Total	12	100.00	

```
. metareg _ES dummy_operation1 dummy_operation2 dummy_operation3 dummy_operation4 dummy_operation5, wsse(_ES) bsest(reml)
Iteration 1: tau^2 = 0

Meta-analysis regression                              No of studies =   12
                                                      tau^2 method      reml
                                                      tau^2 estimate =     0

Successive values of tau^2 differ by less than 10^-4 :convergence achieved
```

	Coefficient	Std. err.	z	P>|z|	[95% conf.	interval]
dummy_oper~1	1.250494	5.911168	0.21	0.832	-10.33518	12.83617
dummy_oper~2	1.937848	5.556943	0.35	0.727	-8.953559	12.82925
dummy_oper~3	4.939995	5.280319	0.94	0.350	-5.40924	15.28923
dummy_oper~4	(dropped)					
dummy_oper~5	1.285008	6.298351	0.20	0.838	-11.05953	13.62955
_cons	-5.269997	5.269997	-1.00	0.317	-15.599	5.059007

Postoperative Systolic Blood Pressure

. tab year, gen(dummy_year)

Year	Freq.	Percent	Cum.
2008	2	20.00	20.00
2011	1	10.00	30.00
2013	1	10.00	40.00
2014	1	10.00	50.00
2015	1	10.00	60.00
2018	1	10.00	70.00
2020	1	10.00	80.00
2021	1	10.00	90.00
2022	1	10.00	100.00
Total	10	100.00	

```
. metareg _ES dummy_year1 dummy_year2 dummy_year3 dummy_year4 dummy_year5 dummy_year6 dummy_year7 dummy_year8 dummy_year9, wsse(_ES) b
> sest(reml)
Iteration 1: tau^2 = 0

Meta-analysis regression                           No of studies =    10
                                                   tau^2 method      reml
                                                   tau^2 estimate =     0

Successive values of tau^2 differ by less than 10^-4 :convergence achieved
```

	Coefficient	Std. err.	z	P>|z|	[95% conf.	interval]
dummy_year1	8.256011	11.32403	0.73	0.466	-13.93868	30.4507
dummy_year2	11.74001	11.07153	1.06	0.289	-9.959786	33.4398
dummy_year3	9.350006	11.18001	0.84	0.403	-12.56241	31.26242
dummy_year4	2.050003	14.25141	0.14	0.886	-25.88224	29.98225
dummy_year5	-1.349991	16.60911	-0.08	0.935	-33.90324	31.20326
dummy_year6	(dropped)					
dummy_year7	8.850006	11.26688	0.79	0.432	-13.23267	30.93268
dummy_year8	6.050003	12.12858	0.50	0.618	-17.72159	29.82159
dummy_year9	5.75	12.25531	0.47	0.639	-18.26996	29.76996
_cons	-11.05	11.05	-1.00	0.317	-32.70761	10.60761

. tab country, gen(dummy_country)

Country	Freq.	Percent	Cum.
China	4	40.00	40.00
India	2	20.00	60.00
Korea	1	10.00	70.00
Mexico	1	10.00	80.00
Thailand	2	20.00	100.00
Total	10	100.00	

Successive values of tau^2 differ by less than 10^-4 :convergence achieved

	Coefficient	Std. err.	z	P>|z|	[95% conf.	interval]
dummy_coun~1	10.20705	12.4777	0.82	0.413	-14.24879	34.66288
dummy_coun~2	9.816587	12.5828	0.78	0.435	-14.84525	34.47843
dummy_coun~3	13.09	12.41918	1.05	0.292	-11.25114	37.43114
dummy_coun~4	(dropped)					
dummy_coun~5	7.258723	12.92236	0.56	0.574	-18.06864	32.58608
_cons	-12.39999	12.39999	-1.00	0.317	-36.70354	11.90355

```
. tab population, gen(dummy_population)
```

Population	Freq.	Percent	Cum.
Adult	5	50.00	50.00
Children	4	40.00	90.00
Unknown	1	10.00	100.00
Total	10	100.00	

```
. metareg _ES dummy_population1 dummy_population2 dummy_population3, wsse(_ES) bsest(reml)
Iteration 1: tau^2 = 0

Meta-analysis regression                                  No of studies =   10
                                                          tau^2 method      reml
                                                          tau^2 estimate =     0

Successive values of tau^2 differ by less than 10^-4 :convergence achieved
```

	Coefficient	Std. err.	z	P>|z|	[95% conf.	interval]
dummy_popu~1	3.425591	2.050994	1.67	0.095	-.5942829	7.445464
dummy_popu~2	(dropped)					
dummy_popu~3	.7153841	3.17111	0.23	0.822	-5.499876	6.930645
_cons	-3.215384	1.950881	-1.65	0.099	-7.03904	.608272

```
. tab operation, gen(dummy_operation)
```

Operation	Freq.	Percent	Cum.
Children oral therapy	1	10.00	10.00
ChildrenOral therapy	2	20.00	30.00
Oral therapy	3	30.00	60.00
Periodontal surgery	1	10.00	70.00
Root canal therapy	1	10.00	80.00
Teeth extraction	2	20.00	100.00
Total	10	100.00	

```
. metareg _ES dummy_operation1 dummy_operation2 dummy_operation3 dummy_operation4 dummy_operation5 dummy_operation6, wsse(_ES) bsest(r
> eml)
Iteration 1: tau^2 = 0

Meta-analysis regression                              No of studies =   10
                                                      tau^2 method      reml
                                                      tau^2 estimate =     0

Successive values of tau^2 differ by less than 10^-4 :convergence achieved
```

	Coefficient	Std. err.	z	P>|z|	[95% conf.	interval]
dummy_oper~1	6.899994	21.07854	0.33	0.743	-34.41319	48.21318
dummy_oper~2	15.29437	18.06463	0.85	0.397	-20.11167	50.7004
dummy_oper~3	15.86448	18.00426	0.88	0.378	-19.42323	51.15219
dummy_oper~4	12.95	18.63337	0.69	0.487	-23.57073	49.47073
dummy_oper~5	(dropped)					
dummy_oper~6	18.58338	17.96318	1.03	0.301	-16.6238	53.79056
_cons	-17.95	17.95	-1.00	0.317	-53.13134	17.23135

Postoperative Diastolic Blood Pressure

```
. tab year, gen(dummy_year)
```

Year	Freq.	Percent	Cum.
2008	2	22.22	22.22
2011	1	11.11	33.33
2013	1	11.11	44.44
2014	1	11.11	55.56
2015	1	11.11	66.67
2018	1	11.11	77.78
2021	1	11.11	88.89
2022	1	11.11	100.00
Total	9	100.00	

```
. metareg _ES dummy_year1 dummy_year2 dummy_year3 dummy_year4 dummy_year5 dummy_year6 dummy_year7 dummy_year8, wsse(_ES) bsest(reml)
Iteration 1: tau^2 = 0
Iteration 2: tau^2 = 3.3858116
Iteration 3: tau^2 = 8.8272995
Iteration 4: tau^2 = 13.411665
Iteration 5: tau^2 = 15.293751
Iteration 6: tau^2 = 15.778457
Iteration 7: tau^2 = 15.883522
Iteration 8: tau^2 = 15.905334
Iteration 9: tau^2 = 15.909821
Iteration 10: tau^2 = 15.910742
Iteration 11: tau^2 = 15.910933

Meta-analysis regression                              No of studies =   9
                                                      tau^2 method      reml
                                                      tau^2 estimate =  15.91

Successive values of tau^2 differ by less than 10^-4 :convergence achieved
```

	Coefficient	Std. err.	z	P>|z|	[95% conf.	interval]
dummy_year1	9.918991	11.27591	0.88	0.379	-12.18139	32.01937
dummy_year2	7.09	11.67091	0.61	0.544	-15.78455	29.96455
dummy_year3	5.539997	12.13003	0.46	0.648	-18.23442	29.31441
dummy_year4	4.639996	12.47748	0.37	0.710	-19.81542	29.09541
dummy_year5	-4.060001	17.94039	-0.23	0.821	-39.22252	31.10252
dummy_year6	(dropped)					
dummy_year7	6.91	11.71462	0.59	0.555	-16.05024	29.87024
dummy_year8	7.940006	11.50033	0.69	0.490	-14.60022	30.48023
_cons	-9.84	10.61775	-0.93	0.354	-30.6504	10.9704

Country	Freq.	Percent	Cum.
China	4	44.44	44.44
India	1	11.11	55.56
Korea	1	11.11	66.67
Mexico	1	11.11	77.78
Thailand	2	22.22	100.00
Total	9	100.00	

```
. metareg _ES dummy_country1 dummy_country2 dummy_country3 dummy_country4 dummy_country5, wsse(_ES) bsest(reml)
Iteration 1: tau^2 = 0

Meta-analysis regression                              No of studies =    9
                                                      tau^2 method       reml
                                                      tau^2 estimate =      0

Successive values of tau^2 differ by less than 10^-4 :convergence achieved
```

	Coefficient	Std. err.	z	P>|z|	[95% conf.	interval]
dummy_coun~1	3.273353	5.594654	0.59	0.558	-7.691968	14.23867
dummy_coun~2	(dropped)					
dummy_coun~3	2.450005	5.882393	0.42	0.677	-9.079274	13.97928
dummy_coun~4	-8.699997	14.84083	-0.59	0.558	-37.78748	20.38749
dummy_coun~5	2.995104	5.438877	0.55	0.582	-7.664899	13.65511
_cons	-5.200005	5.200004	-1.00	0.317	-15.39183	4.991817

[illegible]

```
. tab population, gen (dummy_population)
```

Population	Freq.	Percent	Cum.
adult	5	55.56	55.56
children	3	33.33	88.89
unknown	1	11.11	100.00
Total	9	100.00	

```
. metareg _ES dummy_population1 dummy_population2 dummy_population3, wsse(_ES) bsest(reml)
Iteration 1: tau^2 = 0

Meta-analysis regression                              No of studies =    9
                                                      tau^2 method       reml
                                                      tau^2 estimate =      0

Successive values of tau^2 differ by less than 10^-4 :convergence achieved
```

	Coefficient	Std. err.	z	P>|z|	[95% conf.	interval]
dummy_popu~1	.3513652	2.421951	0.15	0.885	-4.395571	5.098301
dummy_popu~2	(dropped)					
dummy_popu~3	-.3708614	3.390189	-0.11	0.913	-7.01551	6.273787
_cons	-2.52914	1.755954	-1.44	0.150	-5.970746	.9124658

```
. tab operation, gen (dummy_operation)
```

Operation	Freq.	Percent	Cum.
Children oral therapy	1	11.11	11.11
ChildrenOral therapy	1	11.11	22.22
Oral therapy	3	33.33	55.56
Periodontal surgery	1	11.11	66.67
Root canal therapy	1	11.11	77.78
Teeth extraction	2	22.22	100.00
Total	9	100.00	

```
. metareg _ES dummy_operation1 dummy_operation2 dummy_operation3 dummy_operation4 dummy_operation5 dummy_operation6, wsse(_ES) bsest(r
> eml)
Iteration 1: tau^2 = 0

Meta-analysis regression                              No of studies =    9
                                                      tau^2 method       reml
                                                      tau^2 estimate =       0

Successive values of tau^2 differ by less than 10^-4 :convergence achieved
```

	Coefficient	Std. err.	z	P>|z|	[95% conf.	interval]
dummy_oper~1	-14.25	10.78303	-1.32	0.186	-35.38435	6.884346
dummy_oper~2	-6.309998	4.801886	-1.31	0.189	-15.72152	3.101526
dummy_oper~3	-8.054537	5.006087	-1.61	0.108	-17.86629	1.757214
dummy_oper~4	-7.340004	5.294623	-1.39	0.166	-17.71727	3.037266
dummy_oper~5	(dropped)					
dummy_oper~6	-7.69546	5.035655	-1.53	0.126	-17.56516	2.174242
_cons	4.410004	4.410004	1.00	0.317	-4.233444	13.05345

Postoperative Pain Score

```
. tab year, gen (dummy_year)
```

Year	Freq.	Percent	Cum.
2006	1	20.00	20.00
2011	1	20.00	40.00
2020	2	40.00	80.00
2021	1	20.00	100.00
Total	5	100.00	

```
. metareg _ES dummy_year1 dummy_year2 dummy_year3 dummy_year4, wsse(_ES) bsest(reml)
Iteration 1: tau^2 = 0

Meta-analysis regression                              No of studies =    5
                                                      tau^2 method       reml
                                                      tau^2 estimate =       0

Successive values of tau^2 differ by less than 10^-4 :convergence achieved
```

	Coefficient	Std. err.	z	P>|z|	[95% conf.	interval]
dummy_year1	(dropped)					
dummy_year2	.4600003	1.589277	0.29	0.772	-2.654925	3.574925
dummy_year3	1.227098	1.333751	0.92	0.358	-1.387005	3.841202
dummy_year4	-.5599997	2.31106	-0.24	0.809	-5.089595	3.969595
_cons	-1.33	1.33	-1.00	0.317	-3.936753	1.276752

```
. tab country, gen (dummy_country)

    Country |      Freq.     Percent        Cum.
------------+-----------------------------------
     Brazil |          1       20.00       20.00
      China |          4       80.00      100.00
------------+-----------------------------------
      Total |          5      100.00

.
.
. metareg _ES dummy_country1 dummy_country2, wsse(_ES) bsest(reml)
Iteration 1: tau^2 = 0

Meta-analysis regression                              No of studies =   5
                                                      tau^2 method      reml
                                                      tau^2 estimate =     0

Successive values of tau^2 differ by less than 10^-4 :convergence achieved
------------------------------------------------------------------------------
             | Coefficient  Std. err.      z    P>|z|     [95% conf. interval]
-------------+----------------------------------------------------------------
dummy_coun~1 |   1.109972   .6732345     1.65   0.099     -.209543    2.429488
dummy_coun~2 |  (dropped)
       _cons |  -1.209972   .6657662    -1.82   0.069     -2.51485    .0949055
------------------------------------------------------------------------------
```

```
. tab population, gen (dummy_population)

 Population |      Freq.     Percent        Cum.
------------+-----------------------------------
      Adult |          3       60.00       60.00
   Children |          1       20.00       80.00
    Unknown |          1       20.00      100.00
------------+-----------------------------------
      Total |          5      100.00

. metareg _ES dummy_population1 dummy_population2 dummy_population3, wsse(_ES) bsest(reml)
Iteration 1: tau^2 = 0

Meta-analysis regression                              No of studies =   5
                                                      tau^2 method      reml
                                                      tau^2 estimate =     0

Successive values of tau^2 differ by less than 10^-4 :convergence achieved
------------------------------------------------------------------------------
             | Coefficient  Std. err.      z    P>|z|     [95% conf. interval]
-------------+----------------------------------------------------------------
dummy_popu~1 |   .1601602   1.536342     0.10   0.917    -2.851016    3.171336
dummy_popu~2 |       1.23   1.333754     0.92   0.356     -1.38411     3.84411
dummy_popu~3 |  (dropped)
       _cons |      -1.33       1.33    -1.00   0.317    -3.936753    1.276752
------------------------------------------------------------------------------
```

```
. tab operation, gen (dummy_operation)

              Operation |      Freq.     Percent        Cum.
------------------------+-----------------------------------
Pediatric Oral Therapy |          1       20.00       20.00
    Root canal therapy |          1       20.00       40.00
      Tooth extraction |          3       60.00      100.00
------------------------+-----------------------------------
                  Total |          5      100.00

. metareg _ES dummy_operation1 dummy_operation2 dummy_operation3, wsse(_ES) bsest(reml)
Iteration 1: tau^2 = 0

Meta-analysis regression                              No of studies =   5
                                                      tau^2 method      reml
                                                      tau^2 estimate =     0

Successive values of tau^2 differ by less than 10^-4 :convergence achieved

------------------------------------------------------------------------------
             | Coefficient  Std. err.      z    P>|z|     [95% conf. interval]
-------------+----------------------------------------------------------------
dummy_oper~1 |    1.06984   .7755304     1.38   0.168    -.4501717    2.589852
dummy_oper~2 |   -.1601602  1.536342    -0.10   0.917    -3.171336    2.851016
dummy_oper~3 |   (dropped)
       _cons |   -1.16984   .7690562    -1.52   0.128    -2.677162    .3374825
------------------------------------------------------------------------------

.
```
