# Supplementary material for: Efficacy of Music Intervention for Dental Anxiety Disorders: A Systematic Review and Meta-Analysis
Source: Medicina (Kaunas). 2023 Jan 20;59(2):209. doi: 10.3390/medicina59020209 (PMC9961880; doi:10.3390/medicina59020209)
Supplement: Supplementary file 1 [file medicina-59-00209-s001.zip › Supplementary File S5.pdf]

Supplementary File S5: PICOS of the study and the consistency test of the investigators.

PICOS

Review question	Can musical interventions applied to conventional dental treatment be effective in reducing the tension and improving the treatment outcome of patients with dental anxiety?
Population	Patients diagnosed with dental anxiety disorder during previous routine dental treatment or by relevant psychological analysis in the present study.<br>Patients with oral anxiety disorder can exhibit fear, nervousness, resistance to treatment, and even nausea and vomiting, profuse sweating, fainting, and even panic and changes in blood pressure during routine oral treatment.<br>Patients who have received inappropriate oral treatment in the past (especially in childhood) create an unfavorable impression, which in turn leads to resistance and resistance to oral treatment.<br>There are no restrictions on the age, gender, race, or treatment received by the patients participating in the study.<br>Patients were required to have received complete oral treatment and to have undergone preoperative and postoperative testing for relevant vital signs and tension measures.
Subgroups	Subgroup analysis was performed in the study based on the age of the subjects, geographical area, and the items treated.
Interventions	Patients received musical interventions as part of their regular oral treatment, they listened to any type of music in any way during the treatment.
Comparison	Patients received only routine oral treatment, including root canals, extractions, restorations, etc., as needed for treatment
Outcomes	Vital sign-related outcomes: including heart rate, diastolic blood pressure, and systolic blood pressure.<br>Stress-related outcomes: including MDAS, CFSS-DS, and S-AI, etc.<br>Effectiveness of treatment: patient cooperation rate.
Study design	RCTs
Databases	Wanfang, China National Knowledge Infrastructure (CNKI), China Science and Technol-ogy Journal Database (VIP), Pubmed, Web of Science (WOS), ScienceDirect, Cochrane Li-brary, Scopus and CINAHL.

Standard conformance test (K.T and H.L)

**Standard conformance test**		Checker B (H.L)		Total
		Inclusion	Exclusion	
Checker A (K.T)	Inclusion	54	5	59
	Exclusion	10	31	41
	Total	64	36	

Kappa (K.T and H.L)= 0.68
